# Cellular Morphometric Analysis (CellMorph)—a comprehensive imaging‐based tool for quantifying cellular phenotype heterogeneity and dynamics across biological processes

**DOI:** 10.1111/febs.70339

**Published:** 2025-11-18

**Authors:** Henrique Quaiato de Oliveira, Solon Andrades da Rosa, Luiza Cherobini Pereira, Laura Boose de Mendonça, Juliete Scholl, Fabrício Figueiró, Thais Cardoso Bitencourt, Davi Piovesan Echevarria, João Luiz Aldinucci Buzzo, Jessica Boschini D'Agostin, Danieli Rosane Dallemole, Carolina Oliveira, Fernanda Simas, Fernanda Saez‐Calazans, Débora Santos‐Sousa, Guido Lenz, Eduardo C. Filippi‐Chiela

**Affiliations:** ^1^ Instituto de Biociências, Universidade Federal do Rio Grande do Sul Porto Alegre Brazil; ^2^ Programa de Pós‐Graduação em Biologia Celular e Molecular Universidade Federal do Rio Grande do Sul Porto Alegre Brazil; ^3^ Departamento de Bioquímica Universidade Federal do Rio Grande do Sul Porto Alegre Brazil; ^4^ Programa de Pós‐Graduação em Ciências Biológicas: Bioquímica Instituto de Ciências Básicas da Saúde, Universidade Federal do Rio Grande do Sul Porto Alegre Brazil; ^5^ Serviço de Pesquisa Experimental Hospital de Clínicas de Porto Alegre Brazil; ^6^ Departamento de Biologia Celular, Laboratório de Células Inflamatórias e Neoplásicas (LCIN) Universidade Federal do Paraná Brazil; ^7^ Centro de Biotecnologia Universidade Federal do Rio Grande do Sul Porto Alegre Brazil; ^8^ Departamento de Ciências Morfológicas Universidade Federal do Rio Grande do Sul Porto Alegre Brazil

**Keywords:** cell biology tool, cell state, cellular morphometry, phenotypic dynamic, single‐cell biology

## Abstract

Understanding eukaryotic cell morphometry is fundamental to cell biology, as cells exhibit a broad range of sizes and shapes during processes such as senescence, cell death, mitosis, and migration. Dynamic changes in subcellular compartments and protein distribution also occur, impacting cytoplasmic and nuclear characteristics. Traditional measurement methods are often limited, highlighting the need for alternatives that comprehensively integrate data from both the cytosol and nuclei while tracking individual live cells over time. To address these limitations, we developed Cellular Morphometric Analysis (CellMorph), a novel tool designed to objectively assess multiple features of individual eukaryotic cells, including cell size, shape, cytosolic staining, and morphometry. CellMorph can analyze bright‐field and fluorescent images, accommodating both nonstained cells and those expressing fluorescent reporters or chromogenic labels. We validated the tool using various cellular models and specific staining protocols that target fundamental processes such as apoptosis, autophagy, and senescence. CellMorph captures the intricate heterogeneity within cell populations by providing a multidimensional perspective on individual cellular features and their differential responses to various stresses. This capability to track phenotypic changes over time makes CellMorph particularly valuable for studying dynamic cellular responses. Detailed morphometric data are essential for investigating cellular behavior in pathogenic processes and responses to stressors, including therapies or environmental changes. By integrating multiple parameters, CellMorph represents a significant advancement in cell biology, offering researchers a powerful tool to explore the complexities of cellular morphometry effectively.

## Graphical abstract

CellMorph is a groundbreaking tool that allows researchers to analyze multiple features from single cells, including cell size and shape, cytosolic staining and subcellular texture, nuclear staining, and morphometry. By evaluating images from both bright‐field and fluorescence microscopy, it also tracks changes in living cells during key processes such as growth and stress responses, capturing the intricate heterogeneity within cell populations and providing valuable insights into cell behavior, function, and fate.

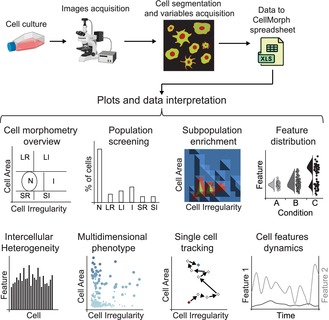

**Abbreviations**
53BP1: p53‐binding protein 1
ATCC: American Type Culture Collection
Bcl‐xL: B‐cell lymphoma‐extra large
BSA: bovine serum albumin
C12‐FDG: 5‐dodecanoylaminofluorescein Di‐β‐D‐galactopyranoside
CII: cellular irregularity index
Cis: cisplatin
CV: coefficient of variation
DAPI: 4',6‐diamidino‐2‐phenylindole
DMEM: Dulbecco's Modified Eagle Medium
EMT: epithelial‐to‐mesenchymal transition
FACS: fluorescence‐activated cell sorting
FBS: fetal bovine serum
FITC: fluorescein isothiocyanate
FSC: forward scatter
GFAP: glial fibrillary acidic protein
GFP: green fluorescent protein
GFP‐LC3: green fluorescent protein tagged with LC3
H2B: histone 2 B
HBSS: Hank's Balanced Salt Solution
LC3: microtubule‐associated protein 1A/1B‐light chain 3
LPS: lipopolysaccharide
LTR: long terminal repeat
NMA: nuclear morphometric analysis
PBS: phosphate‐buffered saline
PEI: polyethylenimine
Rapa: rapamycin
RNA‐seq: RNA sequencing
SA‐β‐gal: senescence‐associated β‐galactosidase
SD: standard deviation
TGF‐β: transforming growth factor‐beta
TMZ: temozolomide

## Introduction

The morphology of eukaryotic cells is a critical indicator of cellular states and behaviors. Both normal and neoplastic cells exhibit a remarkable range of morphometric diversity as they undergo various fates and outcomes, including cell death [1], cell cycle progression [2], mitosis [3], senescence [4, 5], cell differentiation [6], and migration [7]. Information from the cellular shape and size can be associated with specific markers or reporters to assess specific cellular outcomes and fates. Furthermore, cytoplasmic and nuclear changes, including the activity pattern of specific proteins [8], the pattern of subcellular protein distribution [9, 10, 11], or the organization of subcellular compartments [12, 13, 14], can add to the information of cellular morphometry, contributing to the understanding of cellular mechanisms. Additionally, understanding cellular phenotypic dynamics over time allows investigating how cells react to endogenous or exogenous stimuli, providing insights to support the development of strategies for modulating cellular responses and fates [15, 16, 17, 18].

Integrating information from cell morphometry and cytosolic and nuclear features in individual cells can significantly enhance our understanding of cell biology inhuman health and disease [19]. This comprehensive approach sheds light on intercellular heterogeneity by identifying specific subpopulations and their diverse behaviors and can also reflect the natural variability observed in both normal tissues and tumors [20]. Such insights can also contribute to the elucidation of mechanisms involved in disease progression, treatment response, and cellular adaptation [20, 21]. However, while we have observed significant advances in methodologies that integrate gene expression data (e.g., RNA‐seq) in individual cells [22, 23], methodologies integrating multiple phenotypic characteristics in individual cells from microscopy images, including temporal analyses and assessment of cellular outcomes are still scarce [24]. Developing methodologies capable of integrating multiple pieces of information from individual cells from images is essential to allow better mapping and validation of cellular states and phenotypes suggested by gene expression data [25].

Current methodologies for objectively and comprehensively measuring cell size, shape, and morphofunctional characteristics of intracellular compartments are limited and complex, often failing to integrate cytosolic and nuclear data effectively. Moreover, the dynamic nature of cellular morphometry underscores the need for tools that can track individual cells over time, thereby revealing phenotypic heterogeneity and the temporal dynamics of cell states [26, 27]. Therefore, cellular biology studies demand straightforward and objective methods that evaluate eukaryotic cell morphometry alongside additional features, ultimately creating a multidimensional phenotype that captures cells' complex state and behavior with greater accuracy.

Addressing this gap, derived from a previous tool developed by our group, the Nuclear Morphometric Analysis (NMA) [28], here we present Cellular Morphometric Analysis (CellMorph), a novel tool designed for multiphenotypic characterization of individual cells, including features such as size, shape, cytosolic staining and texture, and nuclear morphometry and texture. By providing multidimensional analysis of individual cells from bright‐field and fluorescence images, CellMorph allows the study of cell fate, intercellular heterogeneity, and phenotypic dynamics. We developed and validated CellMorph using various cellular models, with specific staining protocols targeting mechanisms such as apoptosis, autophagy, and senescence. Importantly, CellMorph enables the tracking of phenotypical changes over time, offering valuable insights into the dynamic nature of cell biology and the link between cell responses and cell fate. By integrating diverse morphological parameters, CellMorph represents a significant advancement in the objective analysis of cell states, with potential applications across various biological contexts.

## Results

**Fig. 1:**
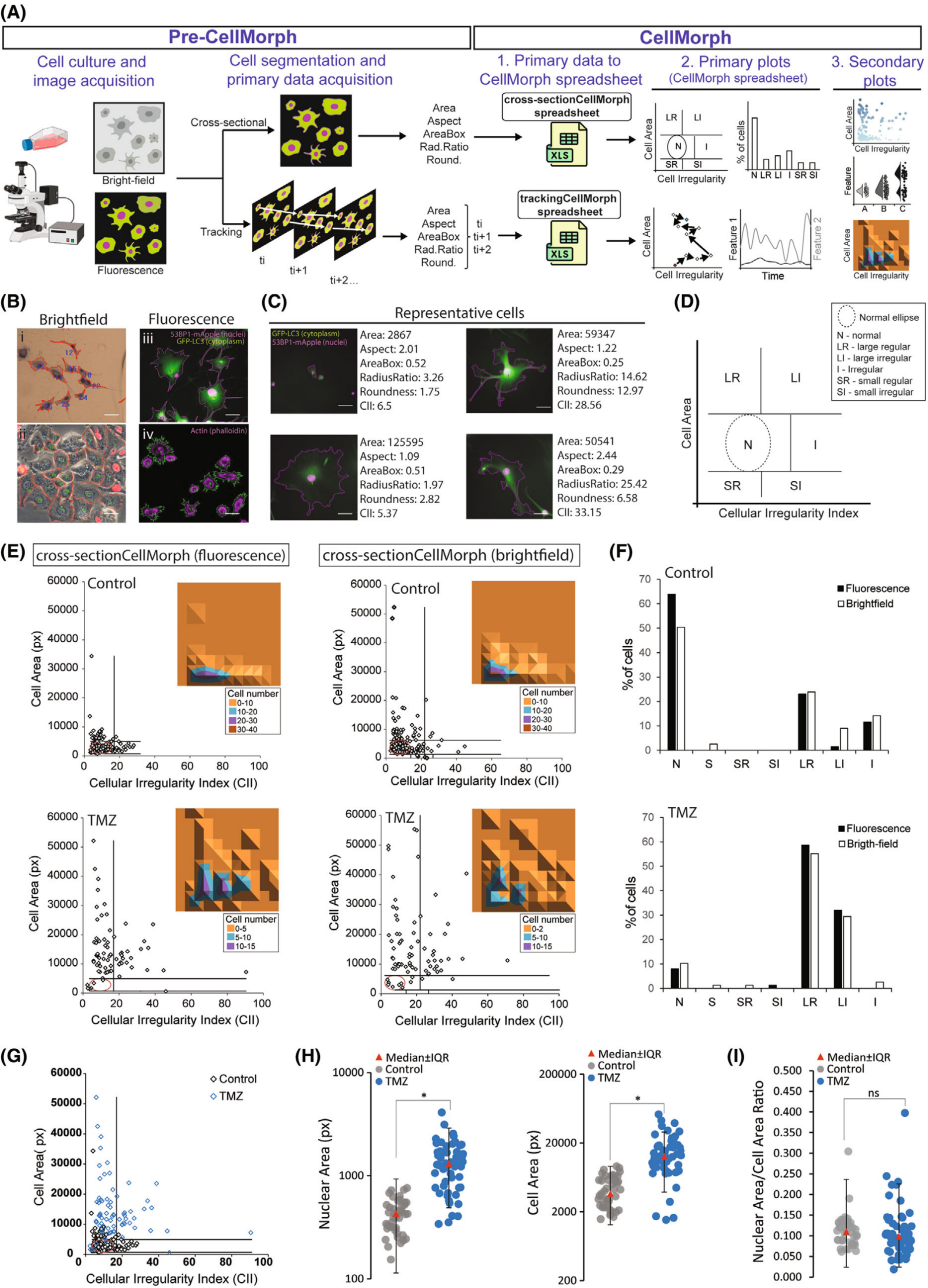
Pipeline and main Cellular Morphometric Analysis (CellMorph) charts. (A) CellMorph pipeline. CellMorph is preceded by image acquisition and cell segmentation, which can be performed in various software programs. The figure shows measurements obtained with Image Pro Plus, the software originally used for the development and validation of CellMorph. After segmentation and primary data acquisition, data analysis and interpretation are performed in dedicated spreadsheets, where primary graphs are generated. Additional graphs (“secondary”) can be generated in other software programs. Images were created using Biorender and Mind The Graph. (B) Representative examples of cell segmentation under bright‐field and fluorescence microscopy; i – control U87 glioblastoma cell line stained with hematoxylin, scale bar: 20 μm; ii – MCF7 breast cancer cell line, scale bar: 20 μm; iii – Temozolomide (TMZ)‐treated U87 cells, scale bar: 20 μm; iv – primary macrophages stained for phalloidin, scale bar: 30 μm. The numbers indicate the label of each cell after segmentation. (C) Representative examples of single‐segmented cells and variables extracted from cellular segmentation and used to calculate the Cellular Irregularity Index (CII); scale bar: 20 μm. (D) Overview of the main CellMorph plot, with regions (quadrants) delimited by user‐defined thresholds. (E) Scatter plots and density plots for control and TMZ conditions for fluorescence‐based CellMorph (data from duplicates, *n* = 130 cells for control, *n* = 74 for TMZ) and bright‐field‐based CellMorph (data from duplicates, *n* = 155 cells for control, *n* = 86 for TMZ). (F) Comparison of CellMorph results obtained from fluorescence and bright‐field images for control and TMZ‐treated cells after 5 days. (G) Composite CellMorph scatter plot for Control and TMZ conditions. (H) Nuclear Area (obtained from nuclear segmentation – Nuclear Morphometric Analysis (NMA)) and Cell Area (cytoplasmic – Cellmorph) for the same cells. Error bars indicate Interquartile Range (IQR). (I) Calculation of the NuclearArea/CellArea ratio for individual cells; data from duplicates, *n* = 42 cells for control, *n* = 54 for TMZ. Error bars indicate Interquartile Range (IQR). *P < 0.05 (Mann–Whitney); ns, not significant. CII, Cellular Irregularity Index; IQR, interquartile range; ns, not significant; px, pixels; Rad. Ratio, Radius Ratio; Round., Roundness; t, time; TMZ, Temozolomide.

CellMorph enables a comprehensive and reliable analysis of multiple phenotypes into a single analytical framework. As for other image analysis pipelines, preliminary image acquisition and cell segmentation steps are required to obtain primary cell shape and size measurements. After that, CellMorph is performed in user‐friendly, semi‐automated spreadsheets designed specifically for graphical plotting and biological interpretation. Therefore, we consider the image acquisition and cell segmentation steps as pre‐CellMorph, as shown in Fig. 1A. Two spreadsheets are available, for cross‐sectionCellMorph (i.e., the analysis of a specific time point, Data S1) or trackingCellMorph (i.e., the dynamic analysis of a single cell, Data S2).

The results section is organized into two main parts: the first part (Protocol for acquiring morphometric measurements and CellMorph spreadsheet use) is divided into two subsections: subsection *Before CellMorph: image acquisition, cellular segmentation, and Cellular Irregularity Index (CII)* briefly describes the main aspects concerning image acquisition and cell segmentation; subsection *CellMorph: step‐by‐step protocols for its use and data interpretation* provides illustrated step‐by‐step protocols for using the CellMorph spreadsheets and data interpretation both for cross‐sectionCellMorph (Data S3) and trackingCellMorph (Data S4). Additionally, we also prepared a video tutorial (Video S1). The second part of the results section describes the validations and illustrates the biological applicability of CellMorph, highlighting its capacity to characterize multiphenotypic features of individual cells.

### Protocol for acquiring morphometric measurements and CellMorph spreadsheet use

Figure 1A describes the main steps of the pipeline, including the image acquisition and cell segmentation that precede CellMorph.

#### Before CellMorph: image acquisition, cellular segmentation, and Cellular Irregularity Index (CII)

Image acquisition can be performed in bright‐field or fluorescence, using a conventional or confocal microscope. Images in .tiff format are recommended to ensure quality. Magnification of 200 to 400× is recommended, but there is no restriction on image magnification, as different cell types vary in size. For single‐cell tracking and phenotypic dynamics analysis, images of the same fields must be acquired, and there is no restriction on the bioimaging equipment used. Likewise, experimental design, including the frequency of images and the phenotypes to be acquired, is a specific variable determined by the user.

Segmentation involves delimiting the cell contour to acquire objective cell size and shape measurements. To develop and validate CellMorph, we utilized the Image Pro Plus 6.0 software, and cell segmentation was performed using the magic wand tool, as demonstrated in the Video S1. Additionally, we tested the execution of CellMorph using measurements obtained through cell segmentation in other software and found similar results, reinforcing the capabilities of CellMorph (Fig. S1). Cells can be segmented from images obtained in bright‐field microscopy, with or without staining (Fig. 1B—left).

Explaining the rationale behind the primary variables underlying CellMorph classification is relevant. The Image Pro‐Plus software offers dozens of area variables and four principal variables for describing an object's shape. Although these four variables can be categorized as shape descriptors, they capture different features of individual cells' shapes. Originally, we performed a principal components analysis (PCA) for dimensionality reduction, aiming to capture the greatest morphometric variance with the fewest variables and obtain two variables (one for area and another for shape) for each cell, allowing for the separation of cells in a scatter plot. The extracted variables in Image Pro‐Plus were Cell Area, Aspect, AreaBox, RadiusRatio, and Roundness. The last four measurements capture different cell shape features as described in Fig. S2A and exemplified in Fig. 1C. Notably, shape variables had medium correlation with each other, as observed for the mean Pearson's correlation of all 20 models used for CellMorph validation and biological applications (Fig. S2B). Based on that, we grouped shape measurements into an index named Cellular Irregularity Index (CII) by summing shape variables whose increase is associated with greater irregularity (Aspect, RadiusRatio, and Roundness) and subtracting those variables whose decrease is associated with greater irregularity (AreaBox). Calculating an index allows capturing the greatest variance for a characteristic (i.e., shape), leading to improved model performance, increased capacity to detect subpopulations, and greater effectiveness in demonstrating data. We also tested the significance of the difference between individual shape variables and the CII in the models used, where it was possible to compare two conditions (e.g., control versus treated, nonpolarized macrophages versus polarized macrophages, control cells versus cells in epithelial‐mesenchymal transition), including both bright‐field and fluorescence images. As shown in Table S1, the difference for CII was, on average, an order of magnitude greater than the isolated variables. Notwithstanding, information on individual cell shape variables can also provide relevant information since they capture and describe different features related to cell shape. Representative examples of these measurements for cells with different morphologies are shown in Fig. 1C. It is also important to mention that there is no correlation between Cell Area and CII in all cell models used for CellMorph development and validation (Table S2).

In addition to cell area and shape variables, the user can also extract other variables of interest for their model that are available in the segmentation software, such as cytoplasmic staining intensity (whether for bright‐field or fluorescence images), texture, staining homogeneity, and others. Throughout the validations section (section CellMorph validations and biological applications), we will provide examples of multidimensional CellMorph graphs that add a third phenotypic feature to each cell in the scatter plot. To construct these multiphenotypic graphs, we used the following python code model:
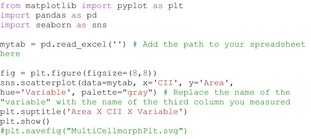


Cell segmentation and variable acquisition can be performed using other software, if variables with similar meanings to those in Image Pro Plus are available. To investigate the versatility of the analysis spreadsheet using primary data from other segmentation software, we segmented the same set of images of U87 cells from control and TMZ‐treated conditions in Image Pro‐Plus, FIJI, CellProfiler, and in our own Python pipeline followed by Napari. The measures with similar meanings extracted in these software are described in Table S3. As shown in Fig. S1, CellMorph scatter plots (Fig. S1A), the values of individual variables (Fig. S1B,C), and the distribution of cells in the CellMorph plot (Fig. S1D) were similar for data generated by the different software, demonstrating that the CellMorph pipeline is functional for primary data obtained from cell segmentation performed in different software programs. Subtle differences observed for some variables are probably due to different cell segmentation methodologies used in each software.

Notably, the process of cell segmentation is the same for cross‐sectionalCellMorph and trackingCellMorph. The differences between these two approaches are in the strategies for image acquisition and subsequent organization of the raw data, and not in the cell segmentation, as illustrated in Fig. 1A.

#### CellMorph: step‐by‐step protocols for its use and data interpretation

The raw data generated by cell segmentation must be analyzed in a specific CellMorph spreadsheet. We provide two spreadsheets, one for performing the cross‐sectional CellMorph (Data S1) and another for trackingCellMorph (Data S2). The rationale for plotting and analyzing data in these spreadsheets is based on flow cytometry analysis strategies, with the creation of gates and thresholds that separate populations from a population of healthy control cells. From this population, quadrants in the graph are determined to categorize cells into different morphometries and additional layers of information (Fig. 1D). The illustrated step‐by‐step process for plotting and interpreting data in the CellMorph spreadsheets, including points of attention and tips, is shown in Data S3 (cross‐sectionalCellMorph) and Data S4 (trackingCellMorph).

### CellMorph validations and biological applications

#### CellMorph in a model of Temozolomide‐induced senescence in glioblastoma cells

For initial validation of the general aspects of CellMorph, from cell segmentation to using the CellMorph spreadsheet and graph plotting, we used the model of glioblastoma cells treated with Temozolomide (TMZ). This model significantly enriches senescent cells [29], which typically present an increase in cell size both *in vitro* and *in vivo* [30]. For this, U87 glioblastoma cells stably expressing the cytoplasmic autophagy marker GFP‐LC3 and the nuclear DNA damage marker mApple^trunc^‐53BP1 were treated with 50 μm TMZ for 5 days, followed by replating in drug‐free medium. After 5 days of treatment ending, cells were imaged in bright‐field and fluorescence to perform the bright‐field cross‐sectionCellMorph and fluorescence cross‐sectionCellMorph. Figs S2C–F, S3 show representative examples of segmented cells in control and TMZ conditions, respectively. A prominent enlargement of cells treated with TMZ compared to control cells can be observed. The heterogeneity between individual cells for the cell shape parameters investigated in CellMorph can also be shown (Figs S2D, S3C). Finally, representative cells with similar cell areas (Figs S2E, S3D) illustrate the features that each shape measurement can capture and the differential capacities to distinguish different cell phenotypes, such as elongated, stellate, or rounded cells.

Biologically, we observed that TMZ led to an increase in the number of large regular (LR) and large irregular (LI) cells, with a concomitant reduction in the number of cells with normal morphometry (Fig. 1E–G). The profile of change in the percentage of cells in each quadrant was similar between CellMorph performed from bright‐field or fluorescence images, demonstrating that the two imaging strategies lead to similar results (Fig. 1F). Furthermore, through density plots it is possible to observe that, while in the control condition, there is only one cellular subpopulation, TMZ treatment led to the emergence of at least two cellular subpopulations with different morphometries (Fig. 1E), demonstrating the potential of the technique in describing intercellular heterogeneity parameters. In addition, Fig. S4A,B illustrate the variation between experimental replicates for cross‐sectionCellMorph using bright‐field and fluorescent images, respectively.

Finally, we also utilized this model to demonstrate the potential of associating CellMorph with nuclear morphometric information obtained from the same cells (images paired) using NMA [28]. Cells used here express both nuclear and cytoplasmic reporters (U87 GFP‐LC3 and mApple‐53BP1trunc), allowing paired imaging of nuclei and cytoplasm of single cells, as described in the step‐by‐step protocols (Dataset S3, S4, Video S1). The full protocol, including the step‐by‐step instructions for NMA, is described in the original article [28] (Fig. 1H). Then, it is possible to calculate the ratio of nuclear and cellular areas for each cell (Fig. 1I), a relevant characteristic in several cellular outcomes as illustrated in the discussion.

#### Cellmorph validation for other cellular models

Given the wide variability of morphometry across cells of different tissue types and origins, we validated the CellMorph pipeline in other cell models to enable broader use. We initially validated the protocol and analysis in four primary glioblastoma cultures (Fig. S5). Fig. S5A shows the overview of the cell morphometry of the primary cultures, both mixed (Fig. S5A—top) and distinguishable by the patient (Fig. S5A—bottom). To further demonstrate the ability of CellMorph to distinguish intertumor cell morphometry heterogeneity, we separated the CellMorph plots for each patient. We analyzed the proportion of cell subpopulations (Fig. S5B) and the mean and variance of cell area and irregularity (Fig. S5C). Our analyses demonstrated that CellMorph can capture the variability of cellular morphologies present in cultures from different patients, which could be associated with other molecular and clinical markers contributing to tumor staging definition, diagnosis, and prognosis.

Finally, we also validated the CellMorph pipeline in two other cell types, breast adenocarcinoma (MCF7 cell line, Fig. S6A—left: two representative images; Fig. S4D shows experimental replicates) and fibroblasts (MRC5 cell line, Fig. S6B—left: two representative images; Fig. S4E shows experimental replicates). Both cell lines demonstrated a similar distribution profile in the CellMorph graph, with more than 70% of the cells presenting normal morphometry. Fig. S6C shows the comparison of the CIIs for different cell models, allowing comparison and demonstrating the variability of shapes for different cell types. It is possible to observe a similarity in the range of values between the glioblastoma cell line U87 in the control condition and the glioblastoma cultures, except the primary culture GB#4, which presented occupation of a larger phenotypic space. Similarly, after treatment with TMZ, we observed an expansion of the phenotypic space occupied by U87 cells, suggesting that the treatment increases cellular morphometric heterogeneity. Finally, we observed that the MCF7 epithelial cell line and MRC5 fibroblasts presented the smallest CII variability intervals.

#### Cellmorph allows the analysis of phenotypic dynamics

**Fig. 2:**
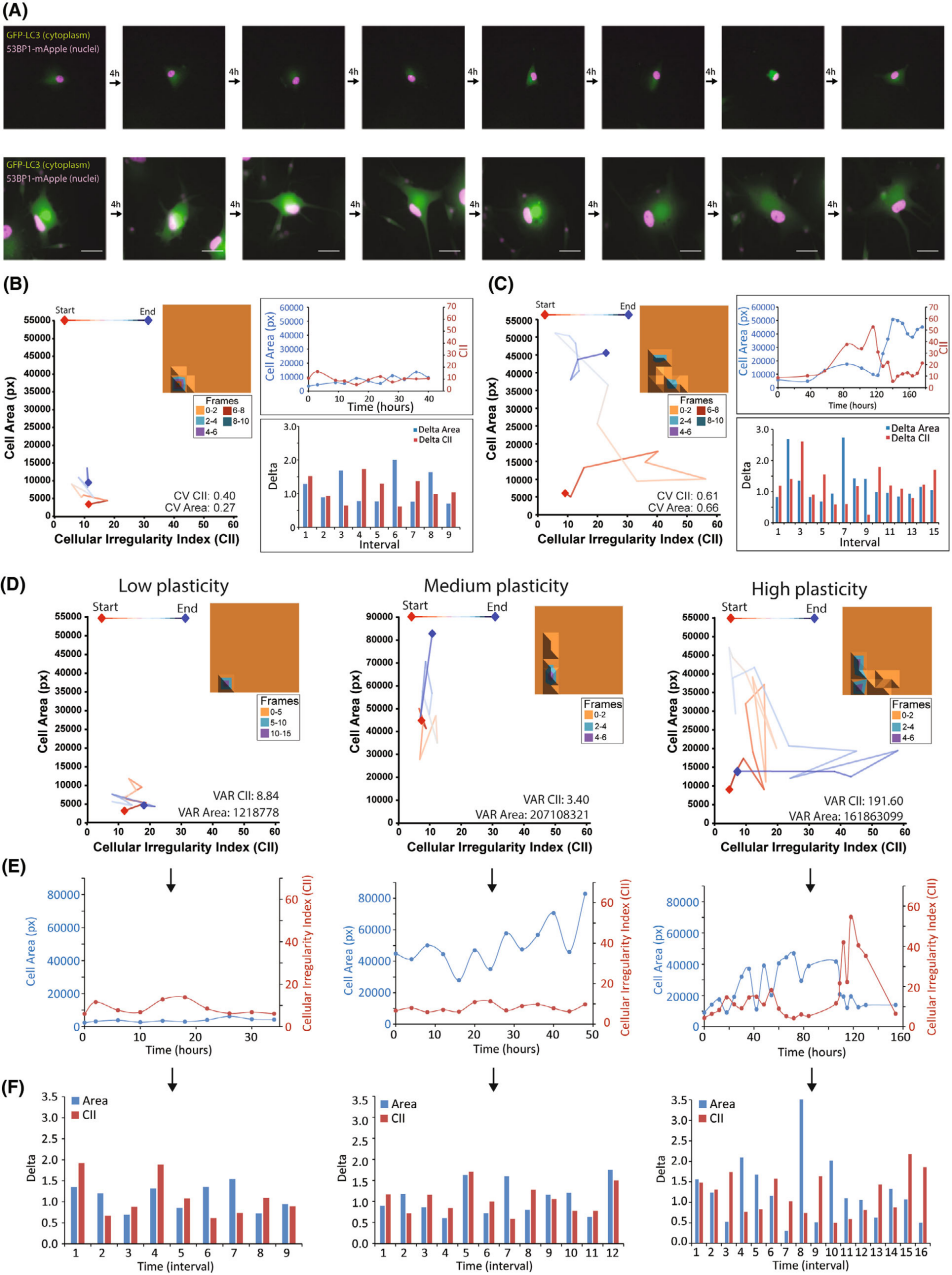
Cellular Morphometric Analysis (CellMorph) can track single‐cell morphometric variations (trackingCellMorph). (A) Representative example of cellular morphometry variation over time of a control and a TMZ‐treated cell; scale bar: 20 μm. (B and C) Representative scatterplots of trackingCellMorph for (B) a control cell and (C) a cell treated with TMZ with a 4‐h interval between each point. Red and blue markers indicate the phenotypic state at the start and end of the track, respectively. The user can also generate density plots (inserts) to assess the most prevalent morphological states of individual cells. Cell Area and CII coefficient of variance (CV) values are also demonstrated. Right graphs in (B) and (C): up – line graphs showing Cell Area and CII over time; bottom – delta values for Cell Area and CII over time. (D) Scatter plots and density plots of representative cells with different degrees of phenotypic plasticity. (E) Line chart of Cell Area and CII over time of the same cells in D. (F) Deltas (variation) of Cell Area and CII over 4‐h intervals, from the same cells displayed in D. CII, Cellular Irregularity Index; CV, coefficient of variance; px, pixels; VAR, variance.

Changes in cell shape and size over time are associated with numerous cellular responses and outcomes. We then advanced a CellMorph pipeline for tracking the phenotypic trajectory of live cells (trackingCellMorph). For this, U87 GFP‐LC3 mApple^trunc^‐53BP1 cells were imaged every 3 h after 5 days of treatment with TMZ 50 μm. A representative example of single cells tracked over time is shown in Fig. 2A, both for control and TMZ conditions. Thus, we obtained the Area and CII information for each time point for each cell, then exported individual cell data to the trackingCellMorph spreadsheet and obtained a graph of phenotypic occupancy over time for individual cells (Fig. 2B,C).

As shown in Fig. 2B, the control cell occupies a limited phenotypic space over time, considering its area and shape. The line and bar graphs shown to the right of the trackingCellMorph plot allow for further analysis of the variability of cell morphometry over time. On the other hand, after the TMZ treatment, there was an expansion of the phenotypic space occupied by cells, as exemplified by the cell shown in Fig. 2C. This greater phenotypic plasticity of TMZ‐treated cells is reflected in markedly higher values of the coefficient of variance (CV) compared to the control cell (see the values at the bottom of the trackingCellMorph plots in Fig. 2B,C). Complementarily, users can calculate other metrics of variability, such as variance or delta (Fig. S7A–C). Finally, considering all cells tracked and segmented over time, trackingCellMorph allows distinguishing cells with low, medium, or high phenotypic plasticity (Fig. 2D–F; see Fig. S7 for other examples). In conclusion, CellMorph can describe the dynamics of morphometric variation of individual cells both under basal conditions and under conditions of environmental changes or pharmacological treatments. Subsequently, the application of trackingCellMorph for analyzing cell death dynamics (Section CellMorph allows estimating mitosis and apoptosis) or epithelial‐to‐mesenchymal transition (Section: Biological applications of CellMorph to epithelial‐to‐mesenchymal transition (EMT) and macrophage polarization) will be described.

#### CellMorph allows estimating mitosis and apoptosis

**Fig. 3:**
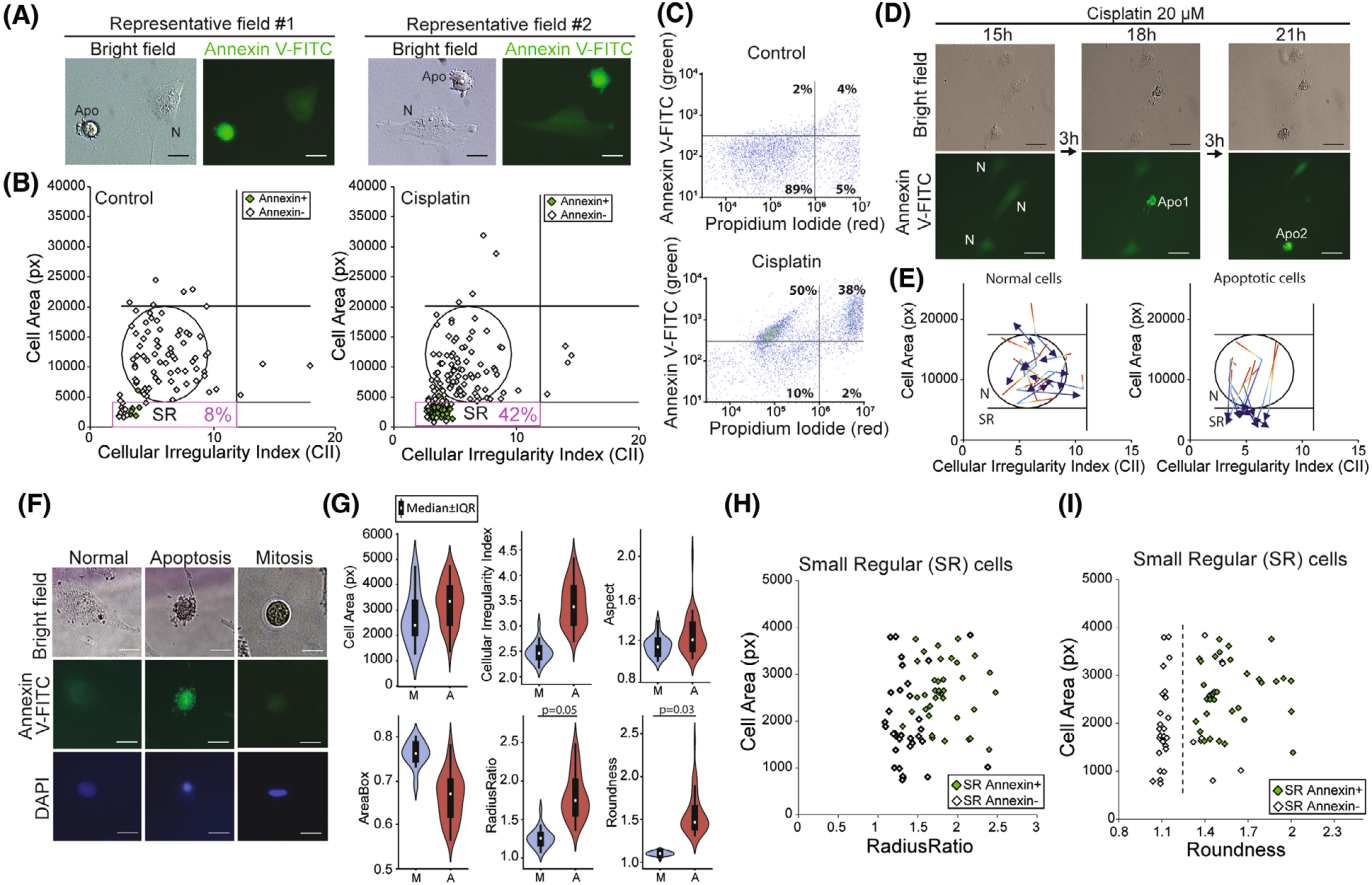
Differential detection of apoptosis and mitosis by CellMorph. (A) Representative examples of apoptotic (Apo) and normal morphology (N) cells under bright‐field and fluorescence (annexin V‐FITC) microscopy; scale bar: 5 μm. (B) CellMorph scatter plot of control and treated (Cisplatin 20 μm) cells; the Small Regular (SR) quadrants are highlighted in magenta, and the percentage of SR cells is numbered. Annexin V‐FITC‐positive cells are shown in green. Data from duplicates, *n* = 96 for control, *n* = 162 for cisplatin. (C) Flow cytometry for Annexin V‐FITC/Propidium Iodide of U87 cells treated with cisplatin 20 μm for 24 h; data from duplicates. (D) Example of individual field tracking after treatment with cisplatin 20 μm under bright‐field microscopy and annexin V‐FITC staining. Images were acquired after 15 h into treatment, every 3 h. N, normal; Apo, apoptosis; scale bar: 10 μm. (E) TrackingCellMorph for normal cells (remaining in the Normal group, N) and apoptotic cells (moving to the Small Regular group, SR). Each tracking starts in red and progresses to blue; the arrows indicate the end point of each tracking. (F) Representative examples of normal, apoptotic, and mitotic cells under bright‐field microscopy, annexin V‐FITC staining, and DAPI (nuclei); scale bar: 5 μm. (G) Comparison between apoptotic (A) and mitotic (M) cells for Cell Area, CII, and primary variables used in CellMorph; data from duplicates, *n* = 71 cells (mitosis = 25; apoptosis = 46). *P*‐values obtained from Mann–Whitney test. (H) CellMorph scatterplots of Cell Area versus RadiusRatio and (I) Cell Area versus Roundness scatterplots using only SR classified cells from B. *n* = 69 cells from two independent experiments. (I) Roundness versus Cell Area scatterplot. Annexin V‐FITC positive cells are colored green. A, apoptosis; Apo, apoptotic cells; IQR, interquartile range; M, mitosis; N, Normal cells; px, pixels; SR, Small Regular.

We then investigated the ability of CellMorph to detect mechanisms associated with cell size reduction. Two mechanisms that exhibit this phenotypic characteristic are apoptosis and mitosis, with potential displacement of cells into the Small Regular (SR) quadrant. To distinguish these two phenotypic states in the CellMorph plot, we exploited two typical changes of apoptosis: the formation of membrane blebbing and the externalization of the phospholipid phosphatidylserine on the cell surface, which can be detected by Annexin V‐FITC staining. Two representative fields containing apoptotic cells (i.e., with membrane blebbing and positive for Annexin V‐FITC) are shown in Fig. 3A. We then enriched the apoptotic cell population by treating U87 wt cells with 20 μm cisplatin for 24 h and performed annexin V‐FITC staining. Thus, we acquired information on each segmented cell's area and shape, in addition to the green fluorescence (mean) information. The SR quadrant was enriched after Cisplatin treatment (Fig. 3B; Fig. S4C shows replicates), to levels like those measured by flow cytometry for Annexin V‐FITC/Propidium Iodide costaining (Fig. 3C). Furthermore, all except one cell positively stained for Annexin V‐FITC located in this quadrant (Fig. 3B), resulting in a higher mean density of Annexin V‐FITC labeling in SR cells than non‐SR cells (i.e., cells from the other quadrants) (Fig. S8A).

Furthermore, we characterized the phenotypic progression of apoptosis based on cell size and annexin staining intensity (Fig. S8B). We observed that initially, cells showed a significant increase in annexin staining, followed by a significant reduction in cell area. Finally, cell area decreased further in the final step of this progression, concomitant with a reduction in annexin staining levels (Fig. S8C). A ROC curve using annexin V‐FITC staining as ground truth for apoptosis generated a value of 0.76 (Fig. S8D).

After that, we performed live cell tracking and applied trackingCellMorph to follow the behavior of cells with normal or apoptotic phenotypes over time, as exemplified in Fig. 3D and Fig. S8E. While cells with a normal phenotype presented an erratic variation of cell morphometry within the normal cell quadrant, cells with an apoptotic phenotype moved throughout the tracking to the SR quadrant (Fig. 3E), confirming the phenotypic trajectory. Notably, cell tracking confirmed that apoptotic cells reduce annexin labeling after cell shrinkage (see the ‘Apo1’ cell in Fig. 3D and the ‘Apo’ cell in Fig. S8E). This observation reinforces the limitation of CellMorph as a pipeline that allows estimating initial apoptosis, when cells have not yet fragmented.

Finally, as mentioned, it is well‐established that, in cell cultures, the process of mitosis also involves cell shrinkage and rounding. However, as shown in Fig. 3F, there is a typical difference between the morphology of cells in mitosis and apoptosis since the latter has a rougher (irregular) surface than the former due to membrane blebbing. We then used differential staining with Annexin V‐FITC to test the capacity of CellMorph to differentiate apoptosis and mitosis. To that, we worked only with SR cells of the original CellMorph graph from Fig. 3B. Considering CellArea, CII, and the primary shape variables, only RadiusRatio and Roundness were significantly different between apoptotic and mitotic cells (Fig. 3G), with a great overlap between them. We then explored the differential capacity of RadiusRatio and Roundness to discriminate Annexin V‐FITC‐positive cells. As shown in Fig. 3H,I, Roundness but not RadiusRatio successfully differentiated Annexin V‐positive cells from Annexin V‐negative cells. Finally, it is noteworthy that, among the cells with greater roundness (those to the right of the dashed line in Fig. 3I), there are some Annexin V‐FITC‐negative cells. This occurs because, as observed in the monitoring of individual cells (Fig. 3D, Fig. S8E), after a few hours of entering apoptosis, the apoptotic cells no longer stain positively for Annexin V‐FITC. It is worth mentioning that other types of cell death besides apoptosis can lead to positive Annexin staining, so it is possible that some cells positively stained for Annexin here are undergoing another type of cell death. Furthermore, it is important to emphasize that we suggest CellMorph as an additional, but insufficient, tool for assessing different types of cell death.

#### CellMorph enables multidimensional analysis of single‐cell phenotype with levels and texture of cytoplasmic markers in individual cells

**Fig. 4:**
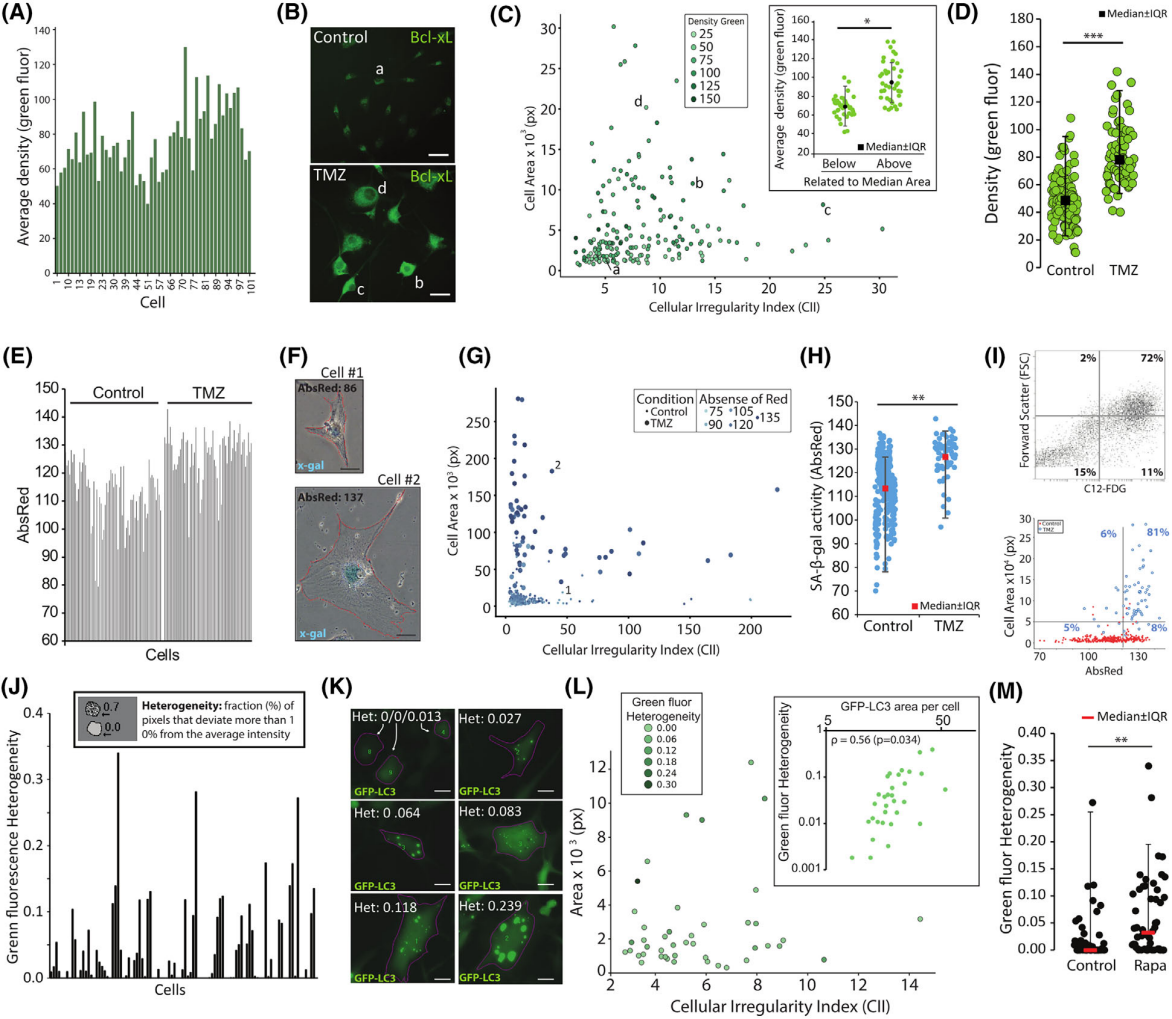
Multidimensional CellMorph allows the analysis of multiple features from individual cells, combining Cell Area and CII with levels and texture of cytoplasmic markers. (A) Variability of green fluorescence levels in 100 cells (U87 MG‐wt) immunostained for Bcl‐xL. Data from duplicates, *n* = 112 for control, *n* = 59 for TMZ. (B) Representative cells immunostained for Bcl‐XL in control and TMZ conditions; scale bar: 20 μm. Letters indicate cells that are shown in the scatter plot in C. (C) CellMorph multidimensional scatter plot integrating data from Cell Area, CII and Bcl‐xL levels in individual cells. Insert: Cells were separated into two groups according to the median area. Then, green density (median) was calculated for cells below and above the median area; *P < 0.05 (Mann–Whitney). (D) Average green density in control and TMZ‐treated cells; data from duplicates, *n* = 121 for control, *n* = 76 for TMZ; ***P < 0.001 (Mann–Whitney); (E) Levels of SA‐β‐gal activity, measured by the Absence of Red color (AbsRed) in 100 representative x‐gal stained cells (U87 MG‐wt). (F) Representative cells stained for x‐gal in control and TMZ conditions; scale bar: 5 μm. (G) CellMorph multidimensional scatter plot integrating data from Cell Area, CII and x‐gal levels (AbsRed) in individual cells. Numbers 1 and 2 indicate two representative cells from F with different phenotypes. (H) Average SA‐β‐gal activity in control and TMZ‐treated cells; data from triplicates, *n* = 297 for control, *n* = 62 for TMZ; **P < 0.01 (Mann–Whitney). (I) Flow cytometry for FSC (cell size) versus C12‐FDG (activity of SA‐β‐gal) compared with Cell Area versus x‐gal (AbsRed). (J) Variability of heterogeneity levels of green fluorescence in 100 cells (A172 GFP‐LC3) cells. Insert: Heterogeneity parameter explained. (K) Representative cells with different levels of cytoplasmic heterogeneity of green fluorescence; scale bar: 20 μm. (L) Scatterplot integrating GFP‐LC3 area data and green fluorescence heterogeneity in individual cells. (M) Average heterogeneity of green fluorescence in control and Rapamycin‐treated (Rapa – 200 nM) cells (A172 GFP‐LC3); data from a single experiment; *n* = 48 for control, *n* = 51 for Rapa; **P < 0.01 (Mann–Whitney). For easier visualization, representative subsets of cells are shown in each panel, while all quantitative analyses were performed using the complete dataset. AbsRed, Absence of Red color; IQR, interquartile range; px, pixels; Rapa, rapamycin; TMZ, Temozolomide.

Thus far, we have validated the CellMorph pipeline and its applicability for distinguishing cellular phenotypes based on cell shape and size. However, including additional features from cytoplasmic staining, considering both levels and subcellular distribution could further increase the applicability and specificity of CellMorph in distinguishing different cellular phenotypes and responses. We then proceeded to develop a multidimensional analysis for individual cells, adding to the information on cell area and shape and features of cytoplasmic staining. Initially, we validated CellMorph to add staining intensity for a cytoplasmic protein as a third piece of information for each cell. For this, we treated glioblastoma cells with TMZ, followed by immunocytochemistry for Bcl‐xL, an anti‐apoptotic protein of the Bcl2 family associated with the survival of TMZ‐treated cells [31]. The variability in green fluorescence intensity between different cells is shown in Fig. 4A, while some representative cells are shown in Fig. 4B. Figure 4C shows a multidimensional CellMorph plot in which the color intensity of each point is relative to Bcl‐xL intensity. Representative cells from Fig. 4B are also indicated. We then separated the cells based on the median area and found that larger cells had higher levels of Bcl‐xL (Fig. 4C—inserted scatter plot). Finally, Fig. 4D demonstrates the increase in Bcl‐xL levels after TMZ treatment, as expected [31].

In addition, we performed a second cytoplasmic staining by measuring the activity of the senescence‐associated acid β‐galactosidase (SA‐β‐gal), which is a standard marker of senescent cells [32]. The enzyme modifies a chromogenic substrate, producing a blue/green product that accumulates in the lysosomes. As recently demonstrated, the best measure for evaluating the intensity of SA‐β‐gal activity is by measuring the absence of red staining in the cell [33]. Thus, in addition to the primary CellMorph measures (i.e., Area, Aspect, AreaBox, RadiusRatio, Roundness), we also extracted, for each cell, the mean red density to allow us to calculate the absence of red staining to estimate SA‐β‐gal enzyme activity for each cell. Figure 4E demonstrates the intercellular variability of the activity levels of 100 representative cells, while Fig. 4F shows two representative examples of cells with the measurements of Absence of Red (AbsRed). Figure 4G shows the multidimensional CellMorph plot, in which the intensity of blue staining of each point is relative to the intensity of SA‐β‐gal enzyme activity measured by the Absence of Red. Smaller points represent the control condition, while larger points represent TMZ‐treated cells. Cells with increased cell area presented higher levels of SA‐β‐gal activity (Fig. 4G). As expected, cells treated with TMZ have increased levels of enzyme activity (Fig. 4H). To validate that, we performed flow cytometry using a fluorescent substrate for SA‐β‐gal. We observed that larger cells (i.e., cells with high Forward Scatter, FSC) present increased enzyme activity levels (Fig. 4I—top; Fig. S9A), corroborating data obtained with CellMorph. Interestingly, the phenotypic patterns of the TMZ‐treated cell population from flow cytometry are similar to that observed in the Cell Area versus AbsRed plot obtained through CellMorph (Fig. 4I—bottom). We also validated the multidimensional analysis of cell morphometry with SA‐β‐gal activity in U87 cells exposed to hypoxia, a context known to induce senescence. As for TMZ, we observed a wide variability in enzyme activity levels between cells (Fig. S9B) and that cells with increased area present more intense levels of SA‐β‐gal activity (Fig. S9C).

Finally, we added the evaluation of cytoplasmic texture to cellular morphometry by measuring the variable ‘Heterogeneity’. As shown in the insert of Fig. 4J, ‘Heterogeneity’, as defined by the Image Pro Plus user guide, represents the fraction of pixels that deviate more than 10% from the average fluorescence intensity. Thus, it is plausible to assume that changing from a diffuse subcellular fluorescence distribution to a punctiform pattern (e.g., vesicle formation) could increase the Heterogeneity value in the cell. To test this, we used a model of U87 cells stably expressing the autophagy marker GFP‐LC3. In these cells, the GFP‐LC3 protein presents a diffuse pattern of distribution throughout the cytoplasm, while it starts to associate with the membrane of autophagosomes creating green dots in the cytoplasm [34]. Figure 4J demonstrates the variability in green fluorescence heterogeneity among individual cells, as exemplified in Fig. 4K. Figure 4L shows a CellMorph plot in which the degree of ‘Green fluorescence heterogeneity’ is indicated in the marker color. This type of analysis of multiple phenotypic characteristics in individual cells can provide evidence to understand better cellular responses and outcomes, such as the crosstalk between autophagy, senescence, and apoptosis in this specific case [35]. Finally, we demonstrated the validity of Heterogeneity as an inference of autophagic levels in this model by observing the significant correlation between green fluor heterogeneity and GFP‐LC3 area in individual cells (Fig. 4L—insert). Corroborating that, we found an increase in the intensity of this measurement after treatment with the classic autophagy inducer Rapamycin (Fig. 4M). It is noteworthy that Heterogeneity as a measure of cytoplasmic texture can be applied to any analysis in which proteins or organelles present a punctiform pattern or punctiform accumulation, such as the measurement of the number of organelles (e.g., lysosomes, mitochondria) or subcellular distribution of other proteins associated with various processes.

#### Biological applications of CellMorph to epithelial‐to‐mesenchymal transition (EMT) and macrophages polarization

Finally, to test the applicability of CellMorph to other cellular processes in which there is phenotypic plasticity associated with morphometric changes, we applied CellMorph to study epithelial‐mesenchymal transition (EMT) and macrophage polarization. EMT is relevant in physiological contexts, such as human development and tissue remodeling, and pathological contexts, such as inflammation and cancer [36]. During EMT, cells shift from an epithelial phenotype to a mesenchymal phenotype, with typical morphometric changes. To validate the use of CellMorph for EMT studies, we selected three published articles using different cellular models and EMT‐inducing stimuli (Fig. S10). Using representative images obtained directly from the articles, we performed cell segmentation and acquisition of variables in images acquired by bright‐field microscopy (Fig. S10A,C,D) and fluorescence microscopy (Fig. S10B), to validate CellMorph for bright‐field and fluorescence images. As shown in the CellMorph plots (Fig. S10E–H), EMT induction led to an increase in the subpopulations of the Irregular (I) and Large Irregular (LI) quadrants (Fig. S10I–L) observed because of the increase in the mean cell area and CII (Fig. S10M–P). CellMorph was also able to show that, after EMT induction, there was an increase in the coefficient of variance of CII, confirming the ability of the method to demonstrate increases in phenotypic heterogeneity and plasticity in cell populations (Fig. S10M–P). Notably, we observed similarity between the percentage of cells in LI and I quadrants of CellMorph and the percentage of cells with mesenchymal characteristics in all EMT models in the articles used as references, reinforcing CellMorph as a tool capable of adding evidence to the characterization of EMT.

Finally, we performed trackingCellMorph using frames extracted from video 1 of the article by Wang et al [37], which showed the induction of EMT in A549 cells treated with TGF‐β (Fig. S11A). After segmenting and plotting the data in the trackingCellMorph spreadsheet, we obtained the phenotypic trajectory of the cell shown in the video (Fig. S11B), which showed an increase in size and CII over time after EMT induction.

**Fig. 5:**
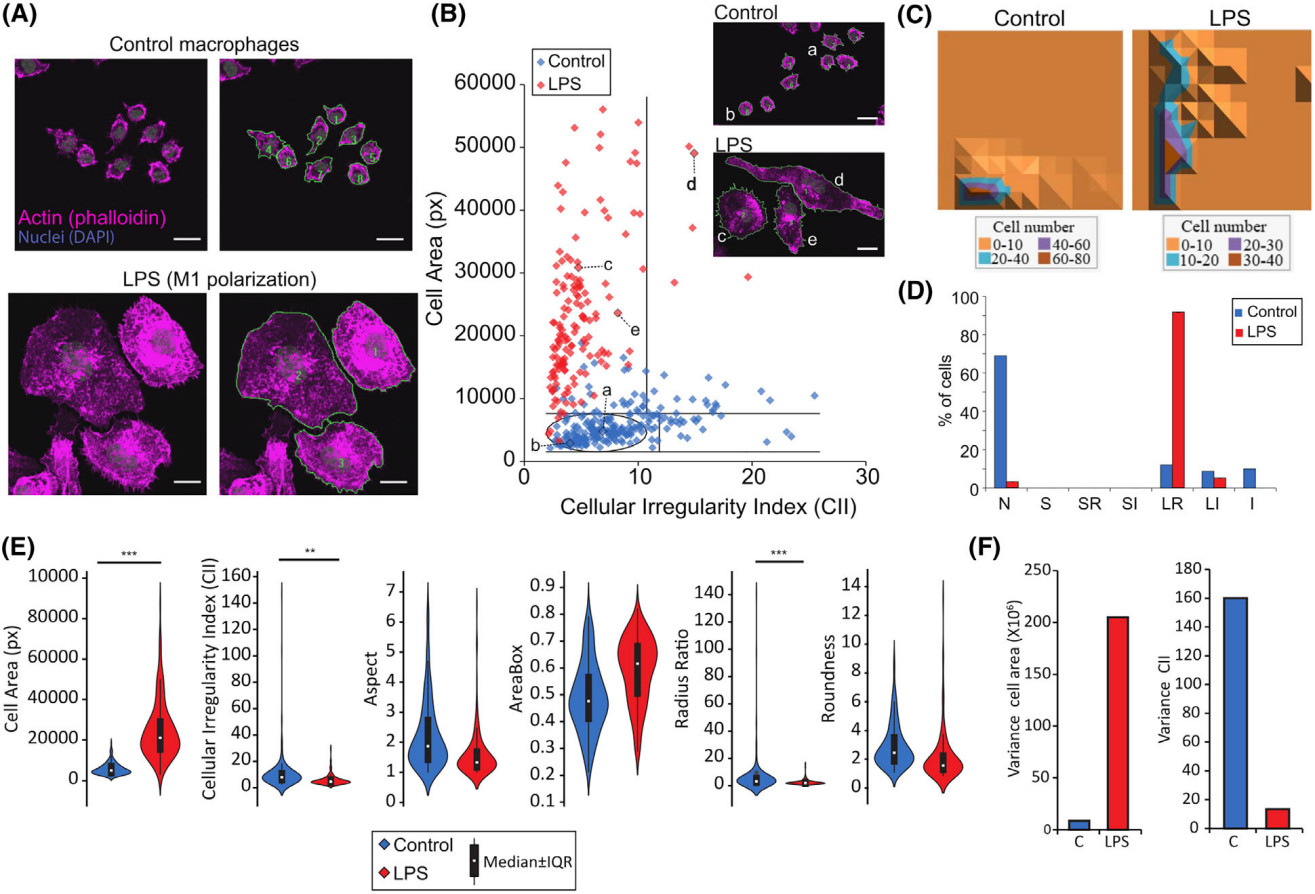
Validation of CellMorph to study macrophage polarization. (A) Representative images of control and LPS‐treated macrophages; scale bar: 20 μm. Cells were treated with LPS followed by staining with phalloidin. (B) Composite CellMorph plot for control and LPS‐treated macrophages; data from a single experiment, *n* = 239 cells for control, *n* = 155 for LPS, from a single experiment. Some representative cells are indicated in the graph and shown in the inserts; scale bar: 20 μm. (C) Density plot for control and LPS‐treated macrophages. (D) Percentage of cells in each quadrant. (E) Violin plots for Cell area, CII and shape variables Average Cell Area and CII to each group, and individual variables of cell shape used to calculate CII. **P < 0.01, ***P < 0.001 (Mann–Whitney). (F) Variance of Cell Area and CII to each group. For C–F: *n* = 239 cells for control, *n* = 155 for LPS, from a single experiment. IQR, interquartile range; LPS, lipopolysaccharide; px, pixels.

We also validated the application of CellMorph to study macrophage polarization since this process involves changes in cell shape and size that could be used as an indicator of macrophage profile [38, 39]. For this, we analyzed the morphometry of macrophages in control conditions and after treatment with LPS [40]. As shown in Fig. 5A, after LPS treatment, macrophages changed from a heterogeneous fibroblastoid phenotype to a phenotype with increased cell size with a regular (round) shape (Fig. 5A). This macrophage activation greatly changed the distribution of cells in the CellMorph scatter plot (Fig. 5B) and density plot (Fig. 5C) and in the quadrants of the graph, with a large increase in the LR population (Fig. 5D). Fig. 5E shows the increase in area and reduction in cellular irregularity, both considering the CII and RadiusRatio (Fig. 5E). The density graph shows that macrophages treated with LPS present greater phenotypic variability considering the cellular area, with the appearance of cellular subpopulations, concomitant with the homogenization of the cellular form (Fig. 5C). Corroborating this evidence, we observed a large increase in area variance and reduction in CII variance after LPS treatment (Fig. 5F).

## Discussion

In this work, we present the development and validation of CellMorph, a simple and objective Cellular Morphometric Analysis method through which it is possible to study multiple characteristics of individual cells, including size, shape, shape patterns, subcellular and cytoplasmic changes, and the dynamics of cell behavior in individual cells over time. We demonstrate that CellMorph can contribute to studies of cell dynamics and heterogeneity both *ex vivo* and *in vivo*, allowing both population screening and profiles of cell subpopulations and the behavior of individual cells. The tool was validated for cell lines of different cell types and primary cultures, considering different responses and mechanisms such as senescence, apoptosis, mitosis, polarization, and epithelial‐mesenchymal transition.

Cellular morphometry analysis tools capable of separating cellular phenotypes into subpopulations with biological relevance and integrating multiple dimensions based on cytoplasmic area, shape, labeling intensity, and texture are rare. Furthermore, available tools typically use complex pipelines or very specific image formats. CellMorph and its derivatives are simple, objective, and low‐cost methods that can be performed from images acquired through bright‐field microscopy (with or without staining) and fluorescence microscopy. Among the main potentialities of CellMorph are (a) the combination of multiple shape variables, each capturing different aspects of cell morphology, in an index that represents a greater percentage of the variance of cell shapes; (b) the definition and validation of quadrants representing subpopulations with phenotypes related to relevant outcomes in cell biology, allowing population screening; (c) the possibility of multidimensional integration of shape, size, intensity of cytoplasmic staining, nuclear morphometry, nuclear and cytoplasmic texture in individual cells; (d) it allows monitoring multiple features of single cells, enabling both the study of cellular outcomes and the dynamics of phenotypic changes; and (e) the possibility of integrating CellMorph with NMA allowing the integration of cellular and nuclear data, contributing to better characterization of complex processes such as development and gene expression [41], cell cycle [2, 42], and cellular senescence [43].

Certainly, one of the most relevant features of CellMorph concerns its multidimensionality. If, on the one hand, single‐cell transcriptomics data have shown an exponential increase in recent years, advances in the multidimensional characterization of cellular and subcellular morphological parameters have been much slower [16]. At least in part, this progress is limited by the need for multidimensional analysis tools for individual cells and the possibility of tracking live cells beyond the biological complexity of some processes. Indeed, the characterization of cellular outcomes such as cell death or cell division requires obtaining evidence from different cellular changes to ensure a specific and accurate diagnosis [44, 45, 46]. For gradual phenotypes such as senescence or plastic phenotypes such as epithelial‐mesenchymal transition, for example, the analysis is even more complex, and the acquisition of more than one piece of information for individual cells improves the accuracy of defining the phenotype [33, 47]. Likewise, considering the countless types of cell death that have been described in recent years, the sum of multiple evidence for individual cells is fundamental to defining the cellular outcome [44, 48]. Furthermore, the advent of single‐cell techniques has demonstrated that even for the same outcome, such as apoptosis or senescence, there is broad heterogeneity [49, 50, 51], and most studies are still based on transcriptomic heterogeneity since cellular morphological analysis tools are scarce. Since many of these phenotypes involve changes in both cellular morphometry and nuclear morphometry, and subcellular compartments or cytoplasm, taking more than one piece of evidence for the same cell through techniques such as CellMorph can allow more precise diagnosis of cellular mechanisms.

Considering trackingCellMorph, using supravital dyes, or constructing cell models that express fluorescent reporters, it may be possible to integrate stress responses to different outcomes in living cells without requiring cell lysis or fixation. In this way, by monitoring the dynamics of cellular morphometry over time, it is possible to investigate phenotypic state transition processes such as quiescence–senescence transition, epithelial–mesenchymal transition, cell migration, cell division, and macrophage polarization. The advent of technologies for monitoring individual cells has revealed that these processes occur in a dynamic and plastic way, with cells going through different phenotypic states in response to different stimuli and inferring association or prediction of cellular outcomes from early responses or phenotypes. For example, it is possible to associate the influence of acute signs of stress, such as levels of DNA damage or autophagy, with differential outcomes, such as apoptosis or senescence [52, 53, 54], levels of stress responses such as autophagy or endoplasmic reticulum stress with different outcomes such as senescence, cell cycle arrest and apoptosis [35, 55], or levels of specific proteins or pathways controlling cell fate such as p21 levels defining the outcome of single cells after chemotherapy [56]. Since CellMorph allows us to obtain cytoplasmic labeling information and can be associated with NMA to obtain nuclear information, analyzing the influence of specific pathways in cell fate can benefit from the construction of cell signaling pathway reporters, such as KTR, which can complement cellular morphometric analyses, providing multifaceted evidence to investigate diverse cellular responses or outcomes [57, 58, 59, 60].

In addition to the mechanisms validated in the manuscript, it is plausible to assume that CellMorph can also be applied to other basic cellular processes, such as the interplay between extracellular matrix and cellular morphometry [7, 61], embryonic development and cell differentiation [62, 63], cell metabolism [2, 43], the dynamics, and heterogeneity of intracellular compartments [64, 65]. Furthermore, while several cellular responses such as cell division, death, motility, and aging occur with dynamic changes in cellular morphometry, in the opposite direction, morphometry itself can also influence gene expression [66, 67], subcellular organelle organization [68], cell fate [69], cell aging [4, 5], and other cellular responses [69, 70, 71], building a crosstalk with great relevance in physiological and pathological contexts. Thus, multidimensional tools such as CellMorph that allow live cell tracking and integration of evidence from acute responses and outcomes can shed light on a better understanding of basic cell biology issues such as how cells control their cellular morphometry [65, 71, 72], the function of cell shape during mitosis [3, 73], the influence of cell size during cell aging [4, 5], or the heritability of cellular morphometry, which can also affect cell fate [74]. Finally, cellular morphometry analyses have also shown applicability in understanding tissue homeostasis and function [75] beyond providing relevant information for prognosis and diagnosis in human diseases [65, 76, 77, 78, 79].

The main limitation of CellMorph is that cellular segmentation in Image Pro‐Plus is semi‐automatic and requires manual work from the user, especially in bright‐field images with no cell staining. Segmenting objects from bright‐field images is a general challenge, which has led to the development of specific bright‐field segmentation tools [80, 81, 82, 83, 84, 85]. However, we still observe enormous limitations, especially considering individualized cell segmentation [82]. Even though it requires manual work, the Image‐Pro Plus object drawing tool overcomes this limitation. This approach prioritizes accurate and precise data by enabling correct cell segmentation, even at the expense of higher productivity. In this scenario, we use a drawing tablet to increase the throughput. Through this, it is possible to perform cell segmentation using a pen instead of the mouse to establish cell segmentation with the same precision or even better. This limitation is strongly reduced when using some cellular dye or fluorescence microscopy imaging, such as CellTracker or others. Furthermore, we validated Cell Morph against other segmenters commonly used by cell biologists, such as FIJI and CellProfiler. The results were quite similar to those obtained from segmentation with Image Pro‐Plus, reinforcing CellMorph's robustness and applicability. Furthermore, other automated cell segmenters for fluorescent images have been developed [86, 87, 88, 89], but these segmenters do not necessarily generate variables identical to those generated by Image‐Pro Plus. Alternatively, it is possible to use plasma membrane markers to perform cell segmentation (e.g., chemical dyes such as DiO or other probes) [90, 91].

In conclusion, CellMorph is a technique that integrates cellular morphometry data with cytoplasmic patterns such as intensity, texture, and dynamics of intracellular compartments and structures in individual cells. In addition to these variables, nuclear and subcellular compartment morphometry and texture data can be integrated into the model, generating a multidimensional cellular phenotyping model applicable to both cross‐sectional and dynamic data in living cells.

## Materials and methods

### Cell lines and models

Human glioblastoma U‐87 MG (ATCC HTB‐14™, RRID:CVCL_0022), human breast adenocarcinoma MCF‐7 (ATCC HTB‐22™, RRID:CVCL_0031), human lung fibroblast MRC‐5 (ATCC CCL‐171™, RRID:CVCL_0440), and mouse macrophage RAW 264.7 (ATCC TIB‐71™, RRID:CVCL_0493) cell lines were purchased from ATCC (American Type Cell Collection, Rockville, MD, USA). From the moment of acquisition, all cells were cultured independently and with specific reagents. Furthermore, all experiments were performed with mycoplasma‐free cells. Cells stably expressing fluorescent proteins (U87 GFP‐LC3 and mApple‐53BP1trunc, MCF7 H2B‐mCherry, MRC5 GFP‐LC3) were all generated through lentiviral transduction. Lentiviral particles were produced by co‐transfecting HEK‐293 cells with the desired reporter plasmid and third‐generation lentiviral vectors (pMDLg/pRRE, pMD2.G, and pRSV‐Rev – Addgene) using linear polyethylenimine (PEI) 25 000 (Polysciences, Warrington, PA, USA; Cat. 23966). Viral‐containing supernatant was collected 4 and 5 days post‐transfection, filtered through a 45 μm syringe filter, and stored at −80 °C. For transduction, cells were plated in 24‐well plates and incubated with 500 μL of the virus suspension along with 8 μg·mL^−1^ polybrene per well, and for cotransduction with two different reporters, 250 μL of each viral suspension was used. Cells were centrifuged at 700 ***g*** for 45 min at 25 °C, and after 24 h, the medium was replaced. Stable cell lines were selected by incubating the cells in medium containing 5 μg·mL^−1^ puromycin (Sigma‐Aldrich, Saint Louis, MO, USA; Cat. P8833) for 3 days, except for MCF7 H2B‐mCherry, which were selected using blasticidin 8 μg·mL^−1^ (Sigma‐Aldrich, Cat. SBR00022). All cells were maintained in DMEM supplemented with 10% Fetal Bovine Serum, 1% penicillin and streptomycin, and 0.1% amphotericin B in 5% CO₂ at 37 °C. The murine macrophage cell line RAW 264.7 was maintained in DMEM High Glucose (Capricorn Scientific, Ebsdorfergrund, Germany; Cat. DMEM‐HPA) supplemented with 1 U·mL^−1^ penicillin, 1 μg·mL^−1^ streptomycin, and 10% heat‐inactivated Fetal Bovine Serum (FBS) (GIBCO, Waltham, MA, USA; Cat. 12657029) in a humidified atmosphere at 37 °C with 5% CO₂ and was subcultured or plated when confluency reached 80%.

### Primary cell culture: sample processing

Primary cell culture was established as previously described [92]. Fresh surgically resected glioma tissues were collected at the Hospital Cristo Redentor, after informed patient consent, between January 2021 and June 2022. A total of four patient cases from both female and male subjects between the ages of 16 and 84 years were included in the present study. The study was approved by the Human Research Ethics Committee of Grupo Hospitalar Conceição (4.418.814) and Universidade Federal do Rio Grande do Sul (3.986.203). Informed consent was obtained for the collection of tissue samples from all patients. The experiments were undertaken with the understanding and written consent of each subject. Tissue was placed in a sterile conical tube containing DMEM/F‐12 (GIBCO) with 10% FBS and 0.5 U·mL^−1^ penicillin/streptomycin (Gibco BRL, USA). The average time from surgical resection to the beginning of the tissue dissociation protocol was 60 min. Briefly, tumor tissue samples were manually minced into approximately 0.5–1 mm diameter pieces using a sterile scalpel followed by enzymatic digestion with collagenase IV (200 U·mL^−1^, Sigma‐Aldrich) and DNAse I (8,  U·mL^−1^) in HBSS buffer for 45 min (37 °C, gentle shaking). Dissociation was stopped by adding FBS. All procedures were conducted in accordance with the standards set by the Declaration of Helsinki ethical principles for medical research involving human subjects.

### Primary cell culture: establishment and characterization of primary tumor cell cultures

The establishment of primary cell culture was performed as described [92]. Briefly, cells were dissociated and resuspended in DMEM/F‐12, 10% FBS, 0.5 U·mL^−1^ penicillin/streptomycin, and amphotericin B. Cells then were seeded in 25‐cm^2^ culture flasks and maintained at 37 °C, humidity 95%, and 5% CO_2_. Media maintenance was performed every two days. Characterization and similarities to the primary glioma tissue were assessed through immunocytochemical staining for GFAP. Briefly, 10^4^ cells were seeded in 48‐well plates, fixed with 4% paraformaldehyde, permeabilized, and blocked with 10% FBS and 0.1% Triton X‐100 for 1 h. Cells were then incubated with the primary antibody anti‐GFAP (1:500, Millipore, MAB360) for 1 h (RT), followed by goat anti‐mouse Alexa‐488 secondary antibody (1:500, Invitrogen, Waltham, MA, USA; Cat. A11001) for 30 min. Nuclei were counterstained with DAPI (300 nm, Sigma‐Aldrich, D9542) for 5 min. EVOS FLoid Imaging System (Thermo Fisher, Waltham, MA, USA) was used for imaging.

### Pharmacological treatments

- Temozolomide (TMZ) (Sigma‐Aldrich, T2577) 50 μm for 5 days, followed by medium removal and growth in drug‐free medium.
- Cisplatin (Cis): Cisplatin (Sigma‐Aldrich, C2210000) 20 μm for 24 h; for single cell tracking concomitant to annexin V‐FITC staining cells began to be imaged 16 h after treatment, every 3 h.
- Rapamycin (Rapa): Rapamycin (Sigma‐Aldrich, 553 210) 200 nm for 24 h.
- Hypoxia: cells were incubated in hypoxia (1% O_2_ + N_2_, 37 °C) for 24 h, followed by 7 days' incubation in normoxia (20% O_2_ + N_2_, 37 °C).

### Cell imaging

All images from each experiment were acquired and analyzed under the same exposure, brightness, contrast, and other settings. Furthermore, all images were acquired ensuring that the fluorescence intensity saturation limit was not reached. Images of U87 GFP‐LC3 mApple^trunc^‐53BP1, including images for live cell tracking, were acquired in an Incucyte^®^ S1 or S3 (Sartorius). Images of primary glioma cells were imaged in Nikon Eclipse TE300. MRC5 GFP‐LC3, MCF7 H2B‐mCherry and images of cells stained for SA‐β‐gal activity with x‐gal or immunostained for Bcl‐XL (Cell Signalling, Lane Danvers, MA, USA; Cat.17229) were acquired in an Olympus IX71 inverted microscope. Images of cells stained for Annexin V‐FITC were acquired in a Zeiss Axiovert 200 microscope. Macrophages imaging was performed using a Nikon A1R MP+ laser scanning confocal microscope (Nikon, Japan) mounted on a Nikon Ti‐Eclipse base.

### Activity of senescence‐associated‐β‐galactosidase (SA‐β‐gal)

#### X‐gal chromogenic staining

Cells were washed in PBS, fixed in 4% formaldehyde for 15 min at room temperature, washed, and incubated with fresh SA‐β‐gal staining solution containing 1 mg·mL^−1^ X‐gal (Sigma‐Aldrich), 80 mm citric acid/sodium phosphate (pH 6.0), 100 mm potassium ferrocyanide, 50 mm potassium ferricyanide, 500 mm NaCl, and 500 mm MgCl for 3 h at 37 °C [32].

#### Flow Cytometry for C12‐FDG

C12‐FDG (5‐dodecanoylaminofluorescein Di‐β‐D‐galactopiranoside) is a substrate for SA‐β‐gal that emits green fluorescence when cleaved by the enzyme. Cells were incubated with 33 μm of (Life Technologies, Carlsbad, CA, USA; Cat. D2893) for 2 h at 37 °C. Stained cells were trypsinized and analyzed using the Attune NxT flow cytometer (Thermo Fisher).

### Annexin V‐FITC staining

Cells were stained for Annexin V‐FITC using the Annexin V Apoptosis Detection Kit following the manufacturer's instructions (Santa Cruz Biotechnology, Dallas, TX, USA; Cat. sc‐4252AK). Briefly, cells were treated with cisplatin at 20 μm for 16 h. After this, cells were washed once with PBS 1x and once with Annexin binding buffer and incubated with a solution containing 2.5 μL of Annexin V‐FITC per sample diluted in PBS 1×. Then, live cells were imaged every 3 h in bright‐field and fluorescence.

### Immunocytochemistry

Cells were plated over a poly‐L‐lysine (Sigma‐Aldrich) 0.01% coated 22 × 22 mm coverslips (Olen, K5‐0015) placed on a 24‐well plate and let proliferate for the determined times. Then, cells were fixed using paraformaldehyde 4% for 20 min at 37 °C. Cells were washed once with PBS 1× and permeabilized with Triton X‐100 0.1%. Protein blocking with 3% BSA was performed for 1 h at room temperature. Primary antibody incubation was overnight at 4 °C, using Bcl‐xL antibody (Cell Signaling, 2764) 1:100. Incubation with a secondary anti‐rabbit IgG labeled with FITC (Santa Cruz Biotechnology), at 1:2000, was performed for 1 h 30 min at room temperature. The slides were mounted with Fluoroshield mounting medium with DAPI (Abcam). Fluorescence intensity was measured using Image J software through the threshold method as described [93]. Total cell fluorescence was obtained through the multiplication of area and MFI variables.

### Macrophage morphology assay

RAW 264.7 cells were seeded on 13‐mm circular coverslips in 24‐well plates at a density of 1 × 10^5^ cells per well. Cells were allowed to adhere for 24 h before being treated with 1 μg·mL^−1^ LPS (Sigma, L2654) or left untreated for an additional 24 h. After treatment, wells were rinsed twice with warm phosphate‐buffered saline (PBS) and fixed in 2% paraformaldehyde (EMS, 15710) in PBS for 20 min. Following fixation, cells were permeabilized with 0.01% saponin (Sigma, S4521) in PBS and stained for 30 min with ActinRed 555 (Invitrogen, R37112) in 0.01% saponin/PBS, according to the manufacturer's instructions. Coverslips were then mounted on glass slides with Fluoromount G containing DAPI (Invitrogen, 00‐4959‐52). Micrographs were captured using 20× and 60× (oil immersion) objectives, with a 405‐nm laser and 450/50‐nm bandpass filter for nuclear staining, and a 561‐nm laser and 595/50‐nm bandpass filter for actin cytoskeleton staining.

### Epithelial‐to‐mesenchymal transition

We validated the applicability of CellMorph for EMP investigation by analyzing images obtained from three different EMP induction models from published articles (original Figs 1D and 5B from [94]; original Fig. 5A from [95]; original Fig. 5 from [96]). Of these references, two are under terms of the Creative Commons Attribution 4.0 International License (http://creativecommons.org/licenses/by/4.0/). For the other reference [94], we obtained the CC license to use the figure in the article.

### Statistical analysis

Samples with normal distribution were analyzed by mean ± SD and Student's *t*‐test or analysis of variance (ANOVA) followed by Tukey test to compare two or more groups, respectively. For samples with abnormal distribution, we used median ± IQR and Mann–Whitney test. Analyses were performed using SPSS 18.0. All *P*‐values under 0.05 were considered significant. In all figures, * indicates *P* ≤ 0.05, ** indicates *P* ≤ 0.01, and *** indicates *P* ≤ 0.001.

## Authors contributions

HQO and SAR contributed equally to the planning and performing of experiments, data analysis, paper drafting, and review and critical intellectual content ECFC contributed as the principal investigator, with funding, lab structure, planning and performing of experiments, data analysis, paper drafting, and review and critical intellectual content. LBM, JS, FF, TCB, DPE, JLAB, JBDA DRD, LCP, FSC, and DSS contributed to the design, acquisition, analysis, and interpretation of data for the work. CO, FS and GL contributed with design, concept, drafting of the work, and reviewing it critically for important intellectual content. All authors contributed to paper reviewing.

## Conflict of interest

The authors declare no conflict of interest.

## Supporting information


**Data S1.** Cross‐sectionCellMorph spreadsheet.

**Data S2.** TrackingCellMorph spreadsheet.

**Data S3.** Step‐by‐step protocol for cross‐sectionCellMorph.

**Data S4.** Step‐by‐step protocol for trackingCellMorph.

**Fig. S1.** CellMorph validation performed using primary data obtained by different segmenters.
**Fig. S2.** Representative examples of CellMorph variables in individual cells in control condition.
**Fig. S3.** Representative examples of CellMorph variables in individual cells after TMZ treatment.
**Fig. S4.** Differential distribution of cells in the CellMorph plot for different experimental replicates.
**Fig. S5.** CellMorph applied to primary culture.
**Fig. S6.** CellMorph applied to MCF7 epithelial breast cancer cells and MRC5 fibroblasts.
**Fig. S7.** Examples of trackingCellMorph.
**Fig. S8.** Relationship between annexin staining and cell size for studying apoptosis.
**Fig. S9.** CellMorph applied to study hypoxia‐induced senescence.
**Fig. S10.** Validation of CellMorph to study epithelial‐to‐mesenchymal transition (EMT).
**Fig. S11.** Validation of trackingCellMorph to study the cellular morphometric variation during epithelial‐to‐mesenchmal transition (EMT).

**Video S1.** Video‐tutorial.

## Data Availability

The data that support the findings of this study are available from the corresponding author echiela@hcpa.edu.br upon reasonable request.
