# Supplementary material for: Cellular Morphometric Analysis (CellMorph)—a comprehensive imaging‐based tool for quantifying cellular phenotype heterogeneity and dynamics across biological processes
Source: FEBS J. 2025 Nov 18;293(14):4185–205. doi: 10.1111/febs.70339 (PMC13370736; doi:10.1111/febs.70339)
Supplement: Supplementary file 4 — Data S4. Step‐by‐step protocol for trackingCellMorph. [file FEBS-293-4185-s001.docx]

**Quaiato and Andrades et al Supp doc. 4**

**Step-by-step protocol for trackingCellMorph**

**Before you begin #1** - organize your raw data in a separate file, which we suggest calling 'Raw Data'. In this file, keep each cell in a different tab (e.g., cell1, cell2, cell3, etc.).

**Before you begin #2** - Download the trackingCellMorph spreadsheet (Supplementary file X).

**Step 1 -** Confirm that you are in the "Normal Cells and Settings" tab (red box). In this tab, the normal ellipse and quadrant definition thresholds will be set.


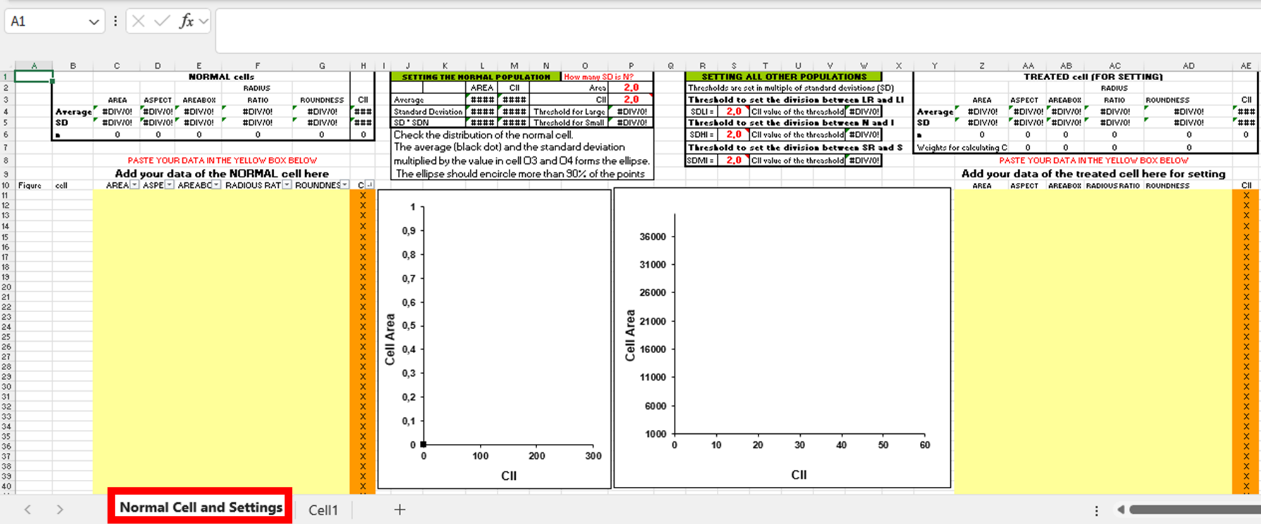


**Step 2** - Copy the raw data from primary measurements (i.e. Area, Aspect, AreaBox, RadiusRatio, Roundness) of all cells from the control condition with healthy morphological appearance.

Attention! At this point, the user must select only cells with a healthy phenotype, not including cells with a phenotype of cell death, senescence, or any other phenotype that differs from the shape and size suggestive of a healthy cell for that experimental condition.

**Step 3 -** Paste the raw data from healthy control cells in the yellow region, starting from position C11 of the spreadsheet (red box). The Cellular Irregularity Index (CII) is automatically calculated in column H (red arrow).


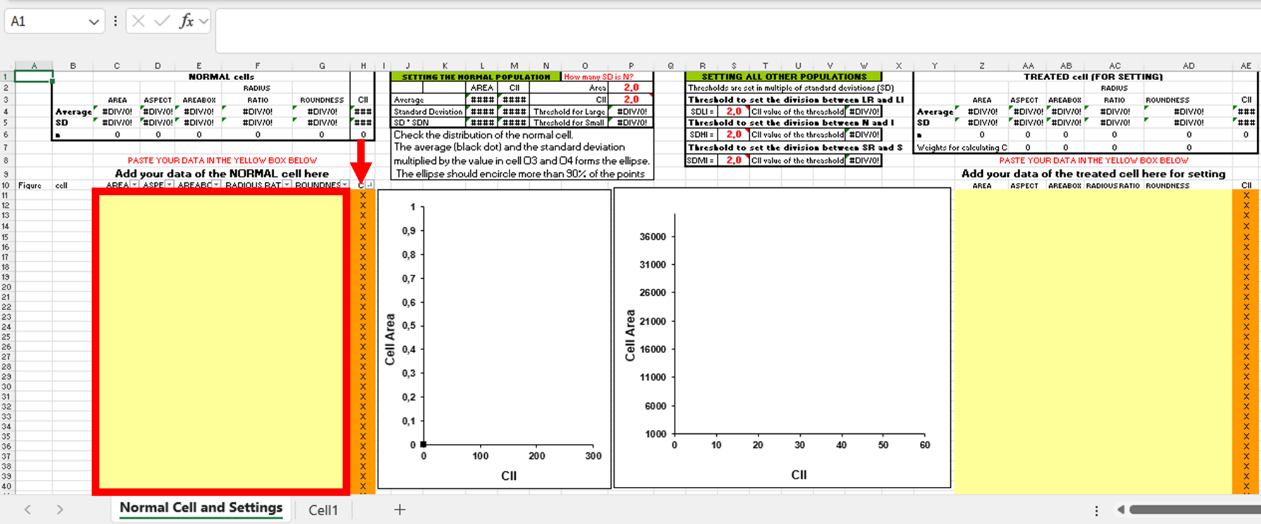


**Step 4** - Column H contains the mathematical formula for automatically calculating the CII (red box). After passing the raw data, you must delete the mathematical formula from all rows where there is no raw data (black arrow) for the scatterplot and the normal ellipse to be set correctly.

Attention! It is essential to delete the mathematical formula from the lines where there is no primary data; otherwise the graph configuration will be incorrect, and it will not be possible to set the normal ellipse that indicates the position of the cell population with a predominantly healthy (normal) phenotype.


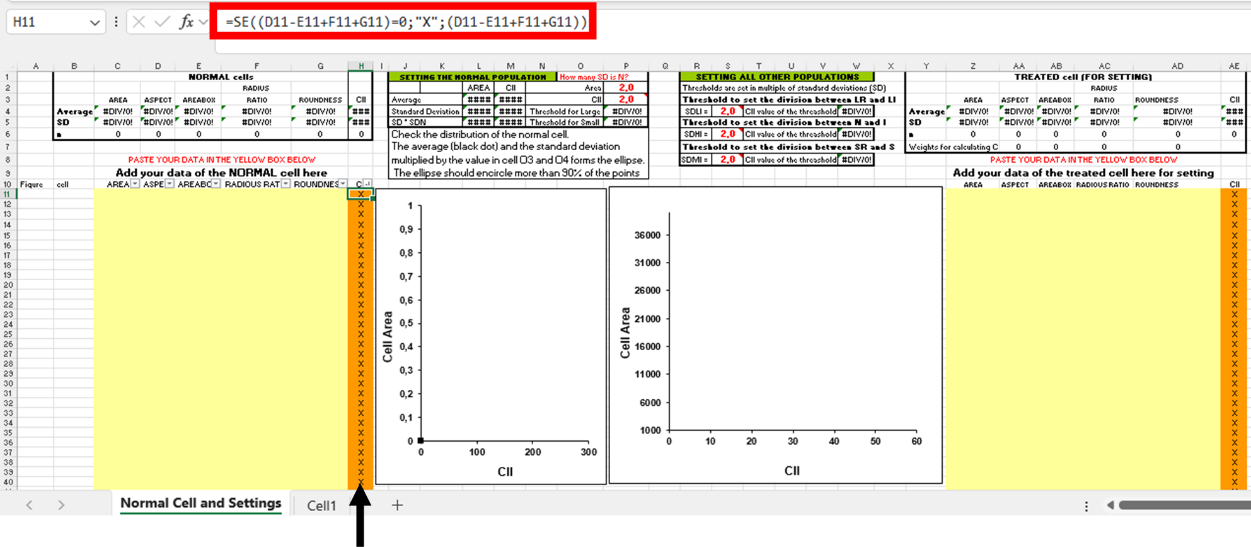


**Step 5** – By transferring the raw data obtained by cell segmentation to the CellMorph spreadsheet, the normal ellipse is automatically plotted, leaving the user to adjust the number of standard deviations. Adjust the number of standard deviations (red arrow) for Cell Area (position P2) and CII (position P3) so that the normal ellipse encompasses at least 90% of the healthy cells plotted in the Cell Area and CII scatterplot (red box). Usually, the number of deviations is between 1.8 and 2.2.


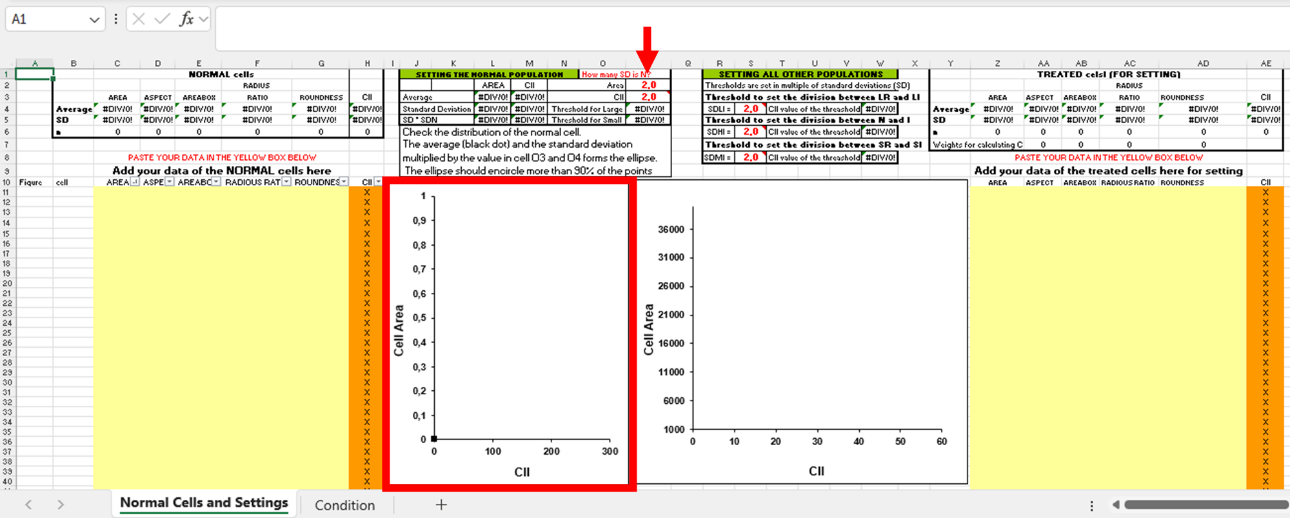


Attention! Correctly adjusting the normal ellipse is critical. The figure below provides examples of adequate, underestimated, or overestimated ellipses. We also suggest critically examining all figures in the article that include scatterplots of Cell Area x CII for the model **(Figure 1E, Figure 1F, Figure 3B, Figure 5B, Figure S5A, Figure S6A-B, Figure S10E-H).**


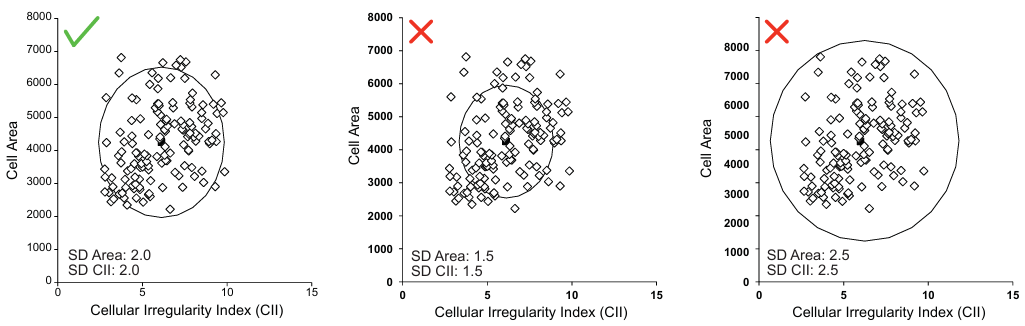
**Normal ellipse setting strategy.** Left: right normal ellipse, built using 2 standard deviations (SD) for Cell Area and CII. Mid: normal ellipse with incorrect setting with a lower than adequate number of standard deviations. Note that many cells are outside the ellipse, underestimating the normal phenotype. Right: normal ellipse with incorrect setting with a number of standard deviations greater than the appropriate number. Note that there is a lot of white space inside the ellipse, overestimating the normal phenotype.

Attention! The percentage greater than 90% for the number of cells contained in the normal ellipse is based on other viability estimation methods, such as flow cytometry or microscopic viability methods (e.g., trypan blue exclusion assay). Therefore, the predominant phenotypes in the control condition are encompassed and classified as Normal, while the remaining 5–10% are distributed in the other quadrants of CellMorph.

Attention! Up to this step, the user aims to set the normal ellipse, which will be the basis for defining the quadrants. From step 6 onwards, the user will start setting the quadrant separation thresholds, still in the "Normal Cells and Settings" tab.

**Step 6** – Paste the raw data obtained from the segmentation of all cells in the yellow region, starting from position Z11 of the spreadsheet (red box).

Attention! Data from ALL cells (i.e. not only control cells) should be pasted here to demonstrate maximum phenotypic occupancy of the graph.


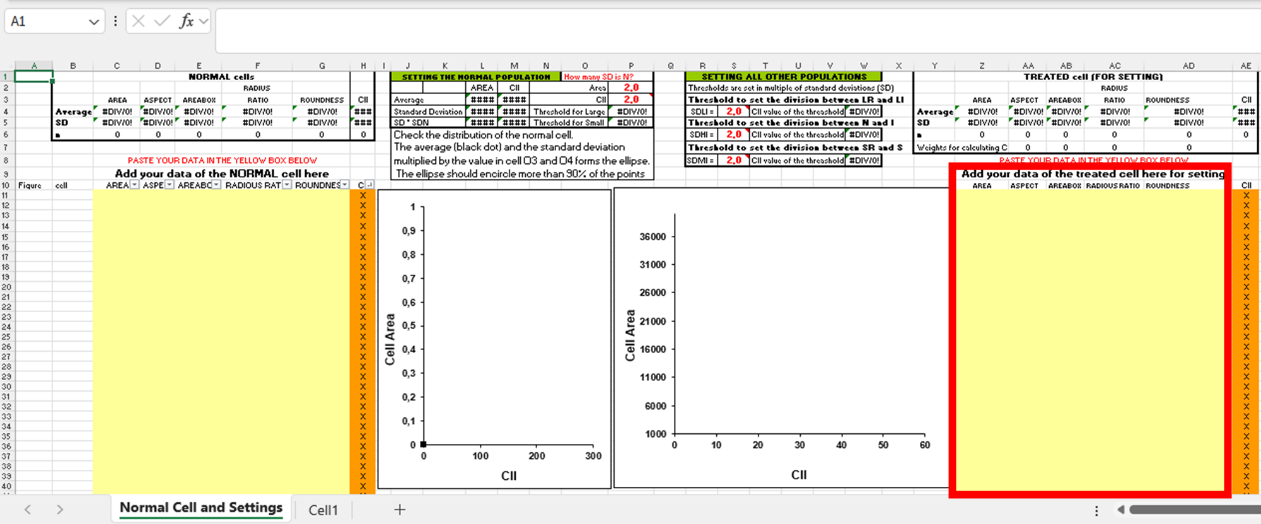


**Step 7** - Just as in determining the normal ellipse, the Cellular Irregularity Index (CII) to each cell is automatically calculated in column AC (red arrow).

Attention! It is essential to delete the mathematical formula (column AC) from the lines where there is no primary data; otherwise the graph configuration will be incorrect and it will not be possible to set the thresholds that define the quadrants.


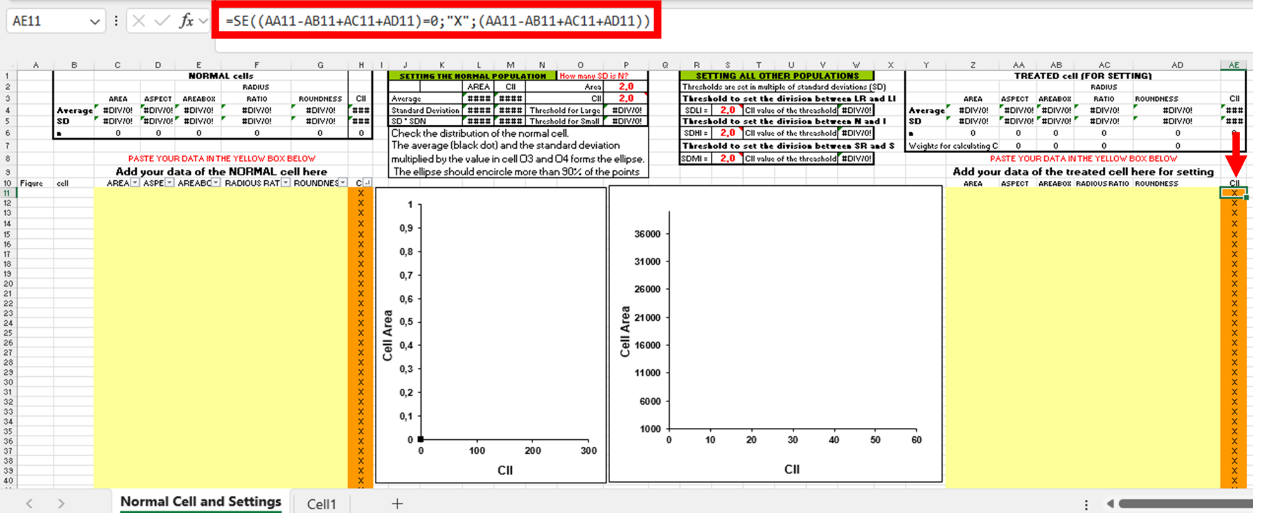


**Step 8** - The user will set the number of standard deviations (red arrow) to determine the vertical thresholds that separate the quadrants of the CellMorph graph (red box). There are 3 thresholds, which separate normal cells from irregular ones (position S6), large regular cells from large irregular ones (position S4), and small regular cells from small irregular ones (position S8). It is essential to delete the mathematical formula from the lines where there is no primary data (red arrow).


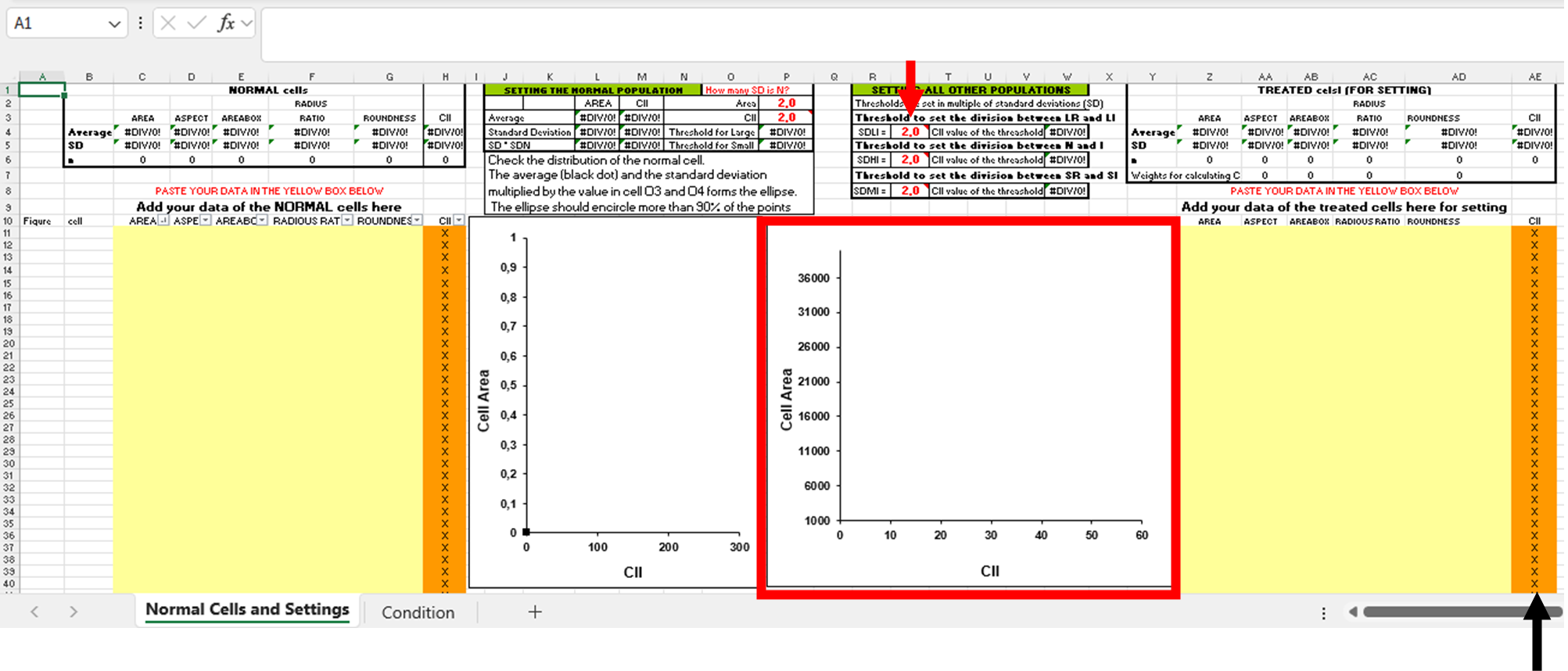


Attention! The horizontal thresholds are automatically determined by the upper and lower limits of the previously set normal ellipse.

Attention! The rationale for determining thresholds comes from flow cytometry gating strategies. The user should enrich the graph with as much data as possible to better define the cell populations and, consequently, the position of the thresholds. Below are shown 2 representative examples of proper threshold determination.


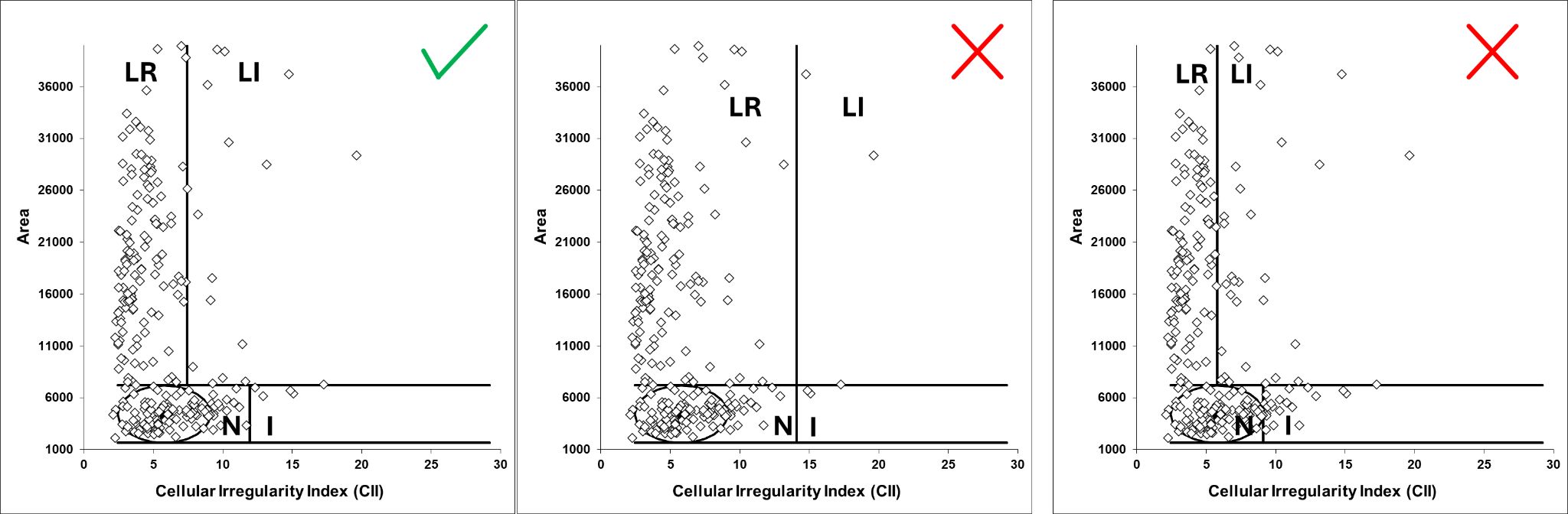


**Thresholds settings - example 1 (LR/LI and N/I).** *Left - Correct thresholds. Thresholds are at the limit of each enriched population in the graph. Middle - Very high LR/LI or N/I separation thresholds, losing the ability to distinguish subpopulations; excessively high thresholds usually leave empty spaces in the quadrant. Right - Very low thresholds, separating cells with similar phenotypes between two distinct populations; thresholds below the appropriate level usually divide cell subpopulations with similar morphometry.*


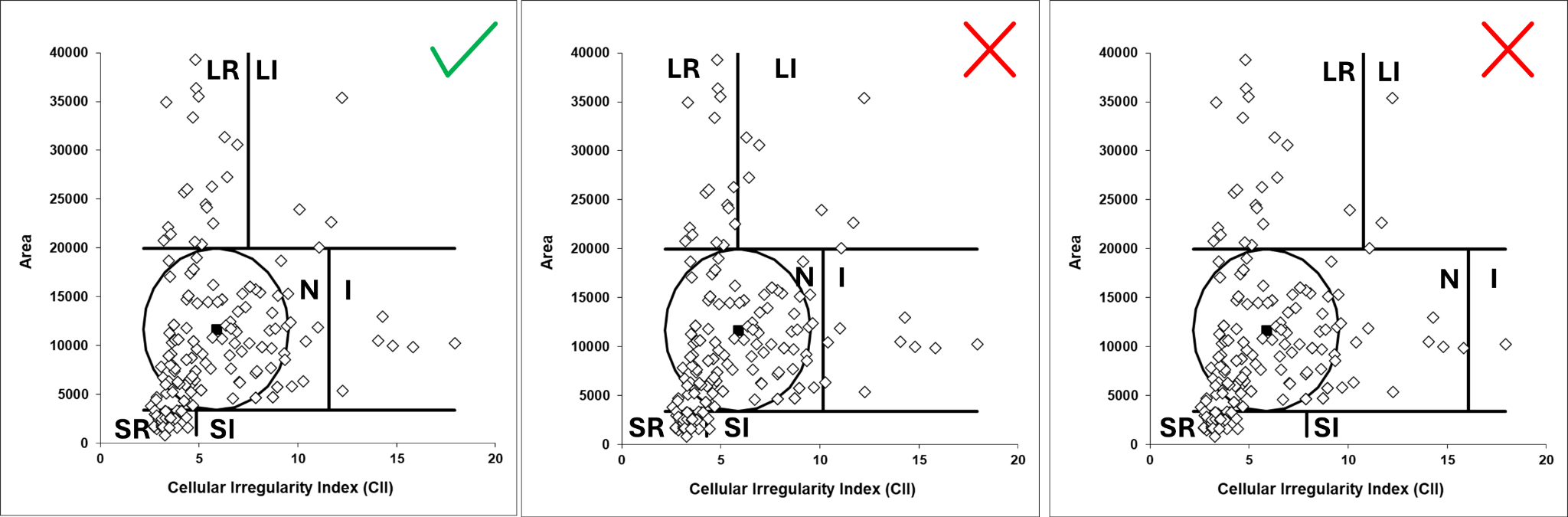


**Thresholds settings - example 1 (SR/SI, LR/LI and N/I).** *Left* - Correct thresholds. Thresholds are at the limit of each enriched population in the graph. *Middle* - Very high LR/LI or N/I separation thresholds, losing the ability to distinguish subpopulations; excessively high thresholds usually leave empty spaces in the quadrant. *Right* - Very low thresholds, separating cells with similar phenotypes between two distinct populations; thresholds below the appropriate level usually divide cell subpopulations with similar morphometry.

Attention! We suggest critically examining Figure 1E, Figure 3B, Figure S5, Figure S6 and Figure S10 in the article that include scatterplots of Cell Area x CII with the thresholds to set the thresholds.

Attention! Up to this step, the user aims to set the normal ellipse and the thresholds, which will be the basis for classifying individual cells in each quadrant (category). Each cell is classified, according to its area and CII, as Normal (N), Irregular (I), Large Regular (LR), Large Irregular (LI), Small Regular (SR) or Small Irregular (SI). From step 9 onwards, the user will perform data analysis to obtain biological evidence.

**Step 9** – Make sure you are in the "Cell" tab (red box).

Attention! The user should not change any other position on this tab. The settings adjusted for normal ellipse and thresholds are automatically applied to the Condition 1 tab.

Attention! The user should duplicate this tab for the total number of cells they have.


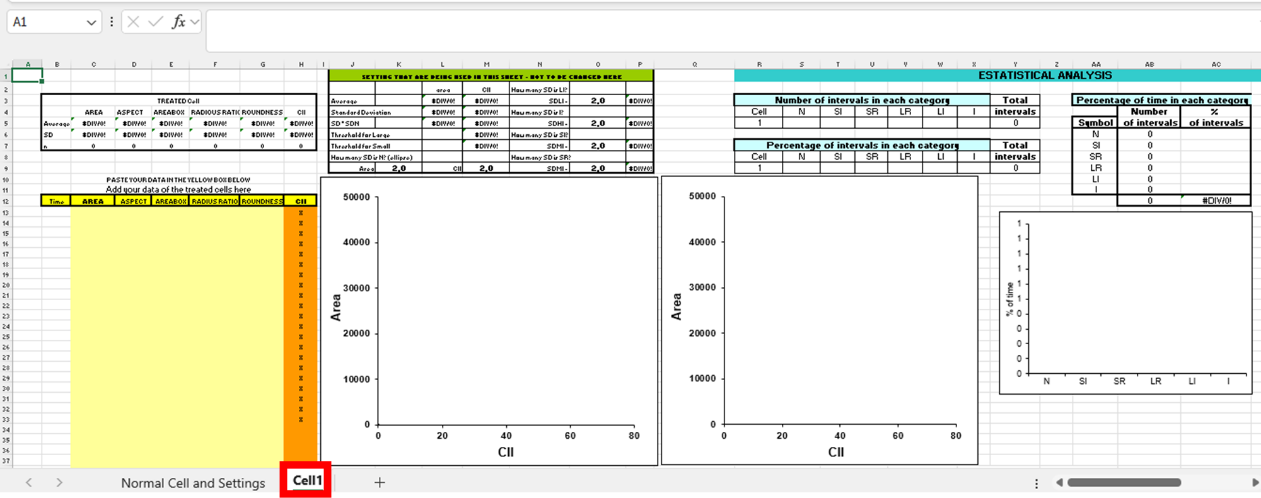


**Step 10** - Paste the raw data obtained from the segmentation of the first cell in the yellow region, starting from position C11 of the spreadsheet (red box).

Attention! The Cell tab is structured for 20 frames as indicated in column B (red arrow).


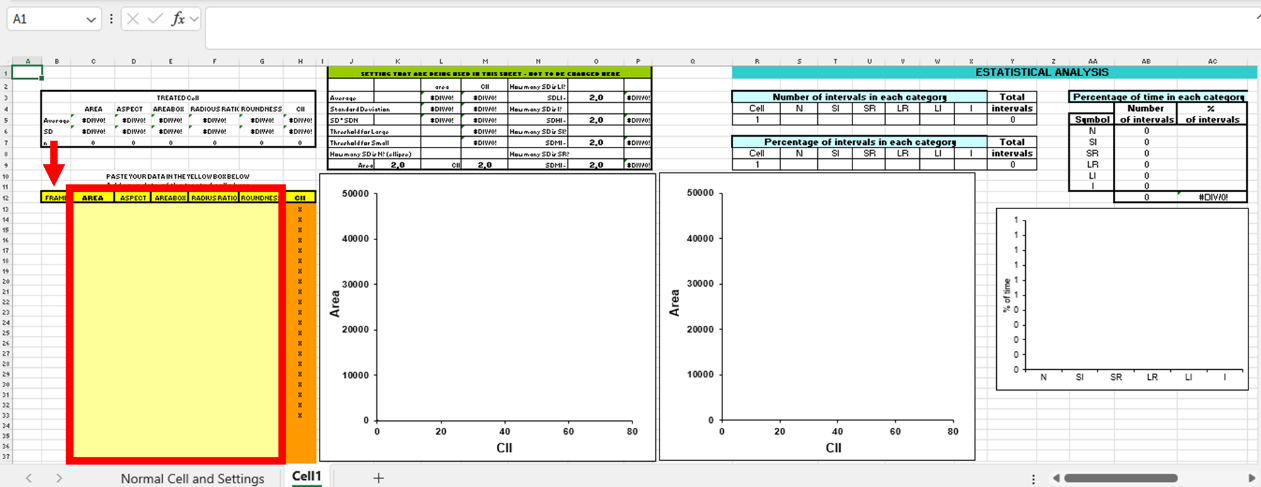


**Step 11** – The Cellular Irregularity Index (CII) to each frame is automatically calculated in column AC (red arrow).


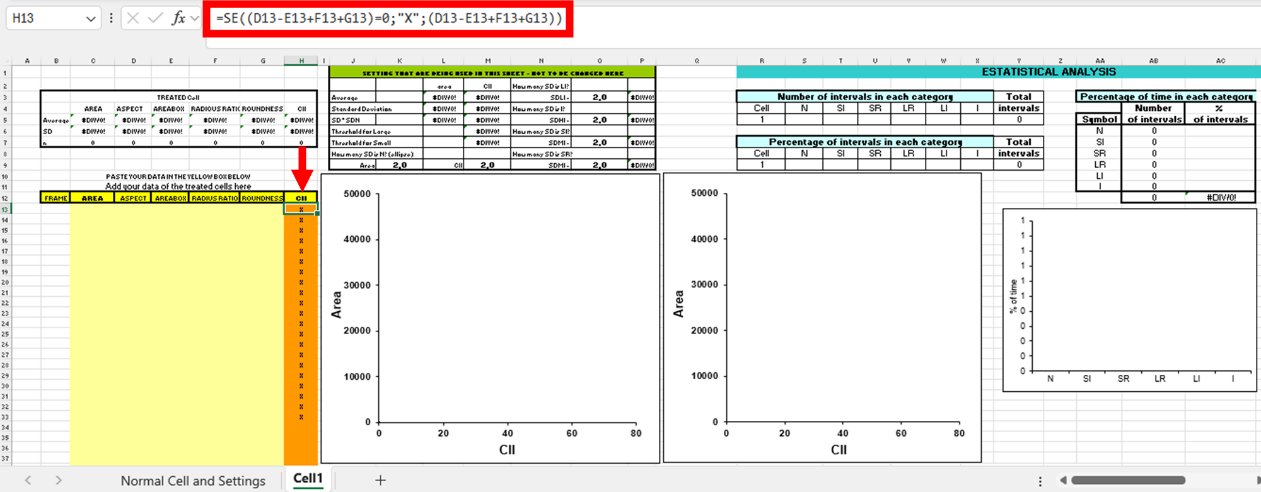


**Step 12** – From this point, the user can obtain biological information. The mean and standard deviation values for Area, Aspect, AreaBox, RadiusRatio, Roundness, and CII are shown in the red box. The number of frames (n) is also shown.

**
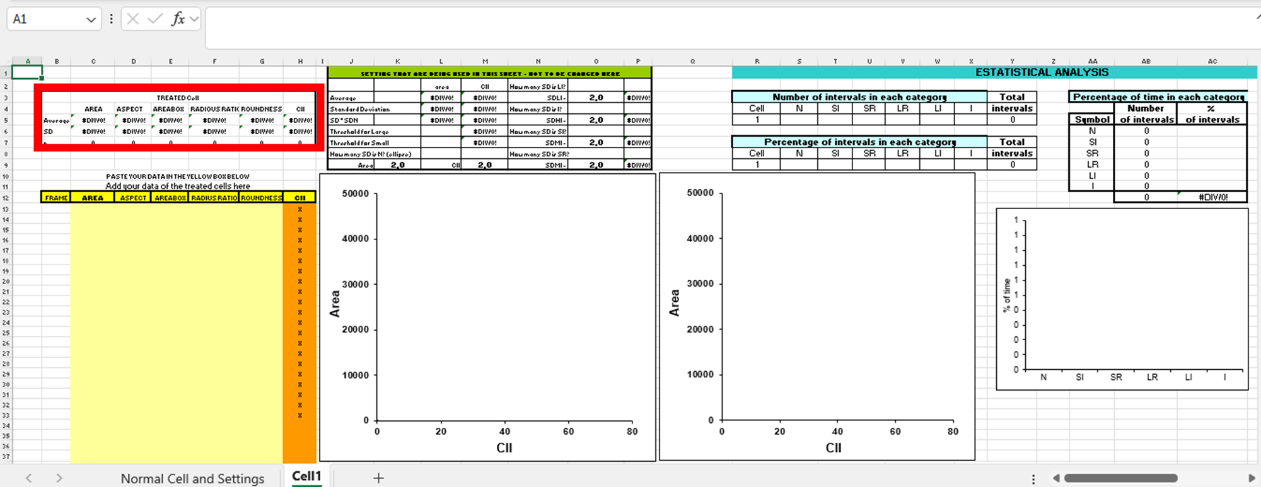
**

**Step 13** – It is essential to delete the mathematical formula (black arrow, column I) from the lines where there is no primary data; otherwise the graph configuration (red and blue boxes) will be incorrect. The graph on the left (red box) is a scatterplot, while the graph on the right (blue box) is a scatterplot connected by lines, describing the cellular phenotypic trajectory.


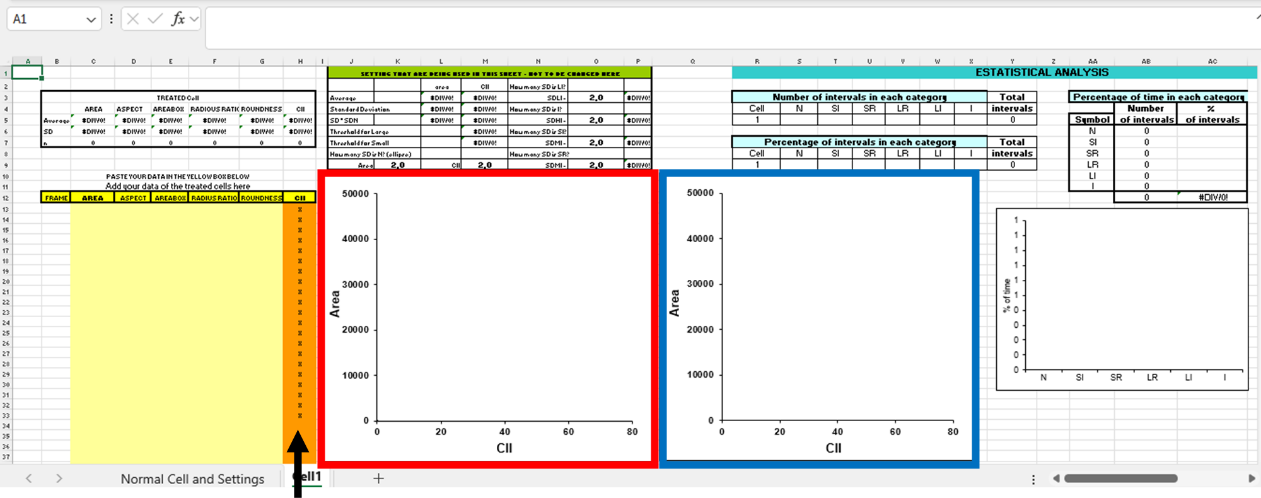


**Step 14** – Red and blue arrows indicate, respectively, the number and percentage of frames during which the cell was in each morphometric category (i.e. N, I, LR, LI, SR and SI).


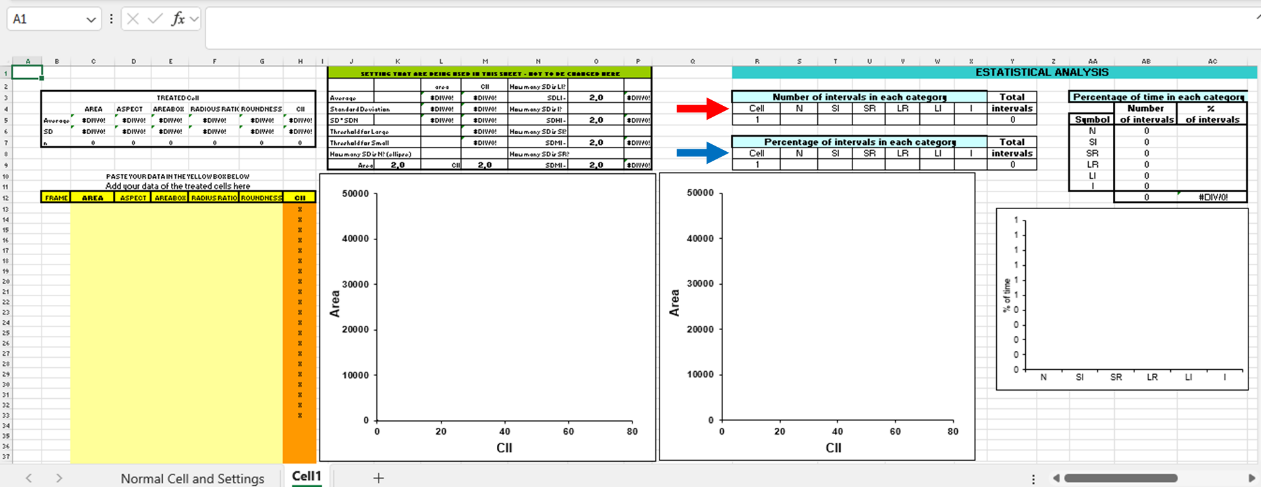


**Step 15** – The spreadsheet also automatically calculates the mean variance and CV for Cell Area and CII over time to the cell. Furthermore, delta values for Cell Area and CII over time are also calculated (blue box).


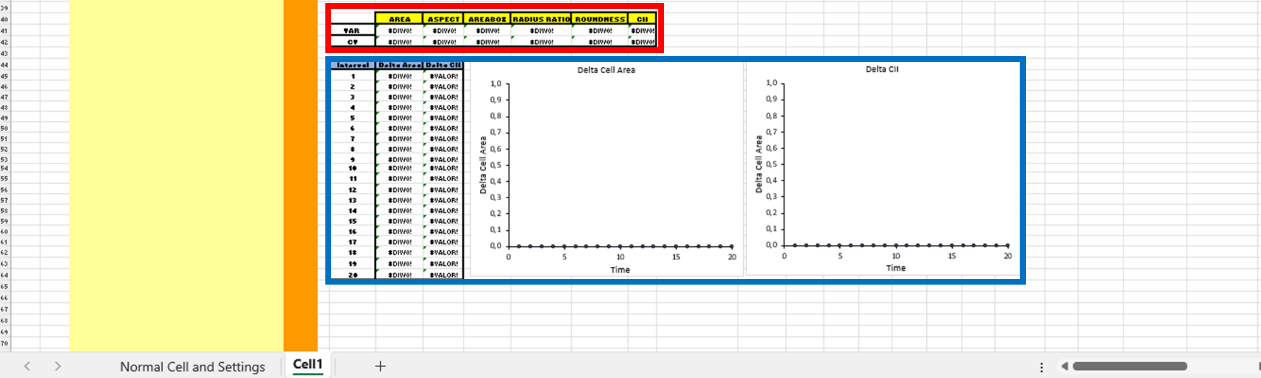


**Step 16** – The number and percentage of frames (intervals) during which the cell was in each morphometric category (i.e. N, I, LR, LI, SR and SI) is organized in the red box. This data is used to build the bar plot (blue box).


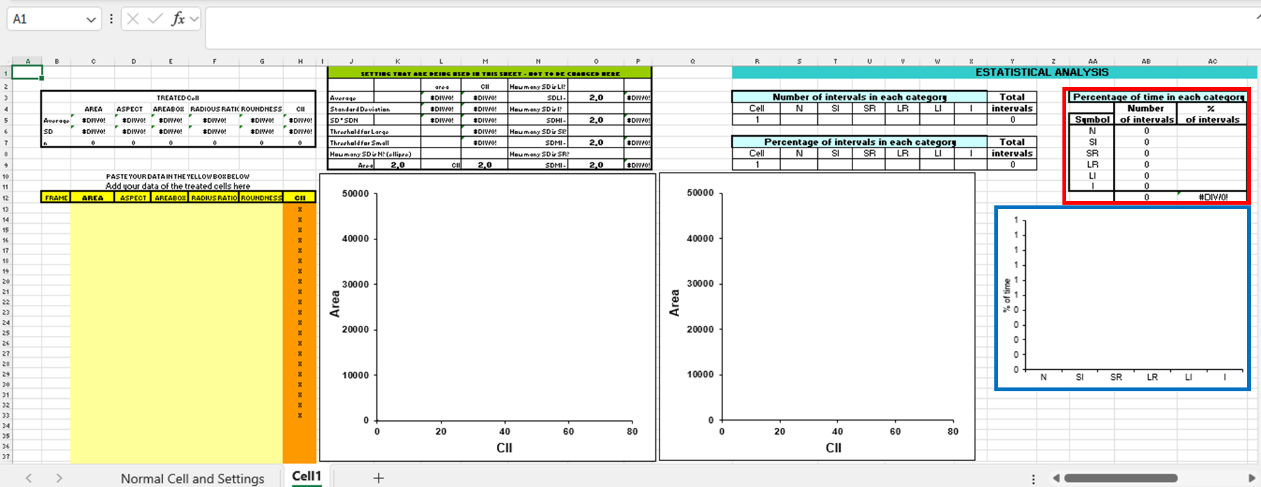


Attention! Users can use primary data (i.e. raw primary data - Cell Area, Aspect, AreaBox, RadiusRatio, Roundness and CII) or secondary data (e.g. the percentage of cells in each category, average Cell Area, average CII, etc.) to plot additional graphs in Excel or other software.
