# Supplementary material for: Cellular Morphometric Analysis (CellMorph)—a comprehensive imaging‐based tool for quantifying cellular phenotype heterogeneity and dynamics across biological processes
Source: FEBS J. 2025 Nov 18;293(14):4185–205. doi: 10.1111/febs.70339 (PMC13370736; doi:10.1111/febs.70339)
Supplement: Supplementary file 5 — Fig. S1. CellMorph validation performed using primary data obtained by different segmenters. Fig. S2. Representative examples of CellMorph variables in individual cells in control condition. Fig. S3. Representative examples of CellMorph variables in individual cells after TMZ treatment. Fig. S4. Differential distribution of cells in the CellMorph plot for different experimental replicates. Fig. S5. CellMorph applied to primary culture. Fig. S6. CellMorph applied to MCF7 epithelial breast cancer cells and MRC5 fibroblasts. Fig. S7. Examples of trackingCellMorph. Fig. S8. Relationship between annexin staining and cell size for studying apoptosis. Fig. S9. CellMorph applied to study hypoxia‐induced senescence. Fig. S10. Validation of CellMorph to study epithelial‐to‐mesenchymal transition (EMT). Fig. S11. Validation of trackingCellMorph to study the cellular morphometric variation during epithelial‐to‐mesenchmal transition (EMT). [file FEBS-293-4185-s005.pdf]

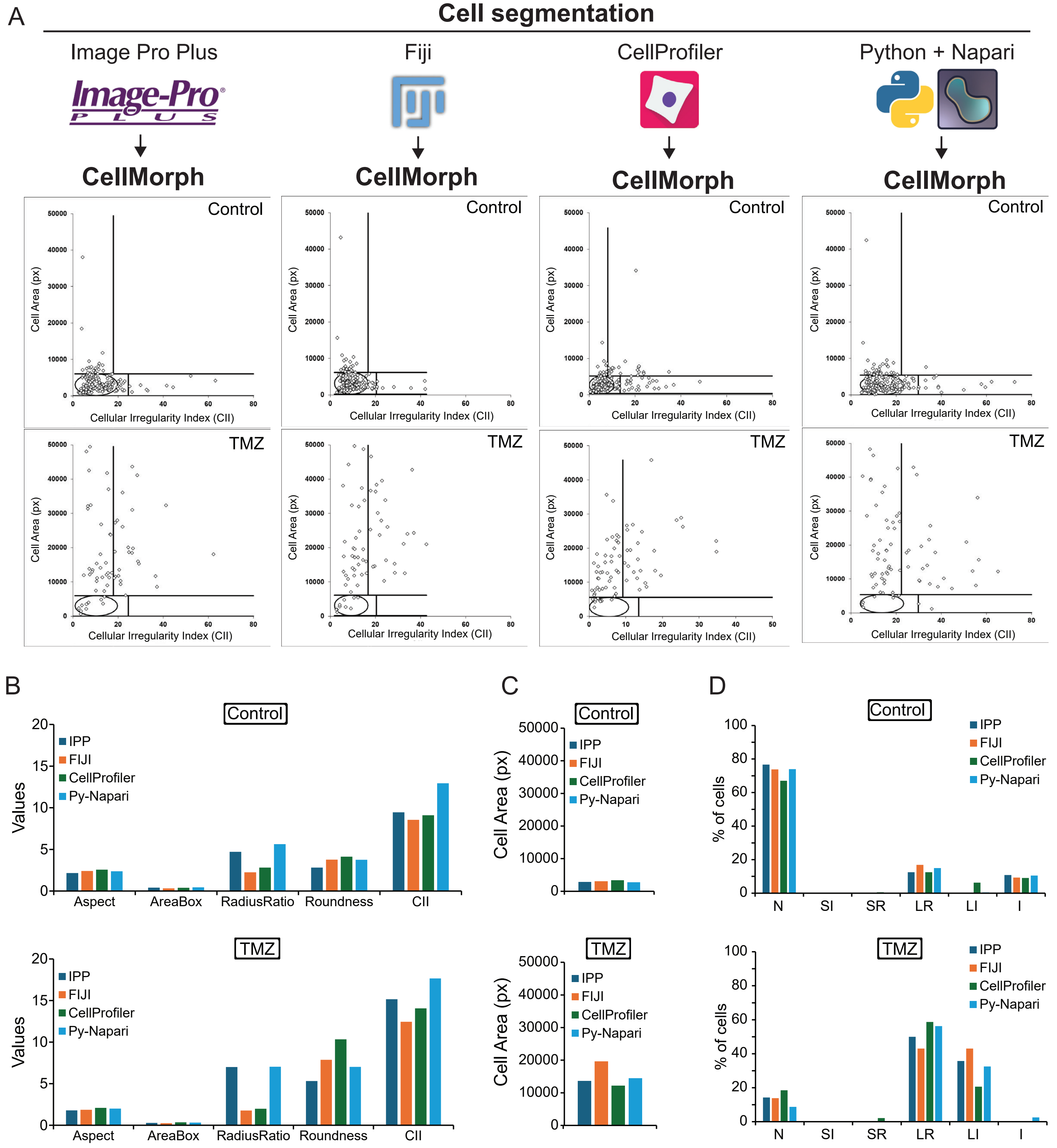

Supp. Figure 1 - CellMorph validation performed using primary data obtained by different segmenters. The same images of U87 cells in the control and temozolomide (TMZ) conditions were segmented. (A) Segmenters used and primary CellMorph graphs obtained for each software. (B) Mean values of the primary shape variables (Aspect, AreaBox, RadiusRatio, and Roundness) and CII for each segmentation software for the control and TMZ conditions. (C) Mean cell area values for each segmentation software for the control and TMZ conditions. (D) Percentage of cells in each category of CellMorph for each software.

A

Aspect

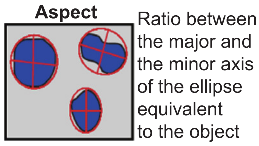

Ratio between the major and the minor axis of the ellipse equivalent to the object

RadiusRaio

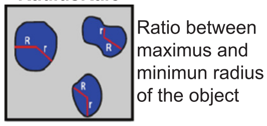

Ratio between maximus and minimun radius of the object

Roundness

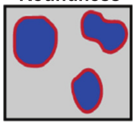

Roundness = (per²)/(4*pi*Area). Roundness of 1 is a perfect circle. Irregular objects have Roundness > than 1

AreaBox

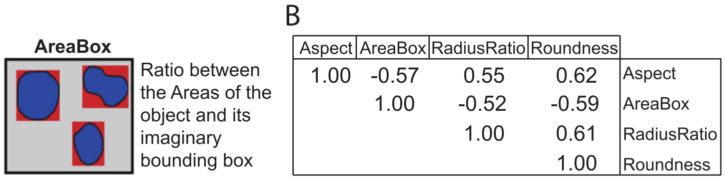

Ratio between the Areas of the object and its imaginary bounding box

B

Aspect	AreaBox	RadiusRatio	Roundness	
1.00	-0.57	0.55	0.62	Aspect
	1.00	-0.52	-0.59	AreaBox
		1.00	0.61	RadiusRatio
			1.00	Roundness

C

Control Cells

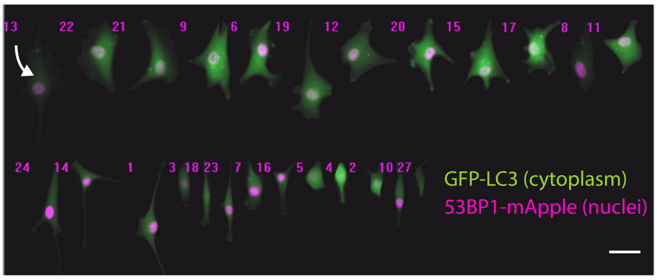

* magenta numbers represent the cell label in the Image Pro Plus output; the cell's label corresponds to the number to its left, as exemplified in the first cell.

D

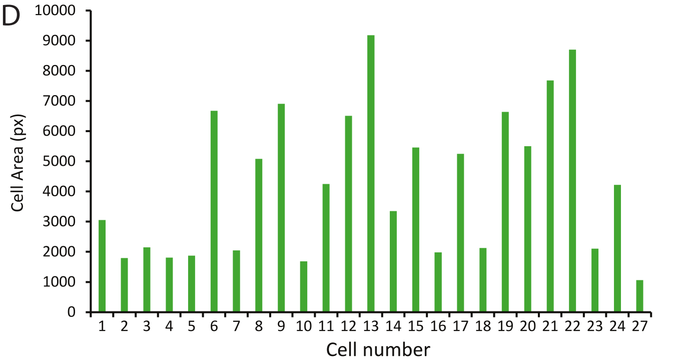

E

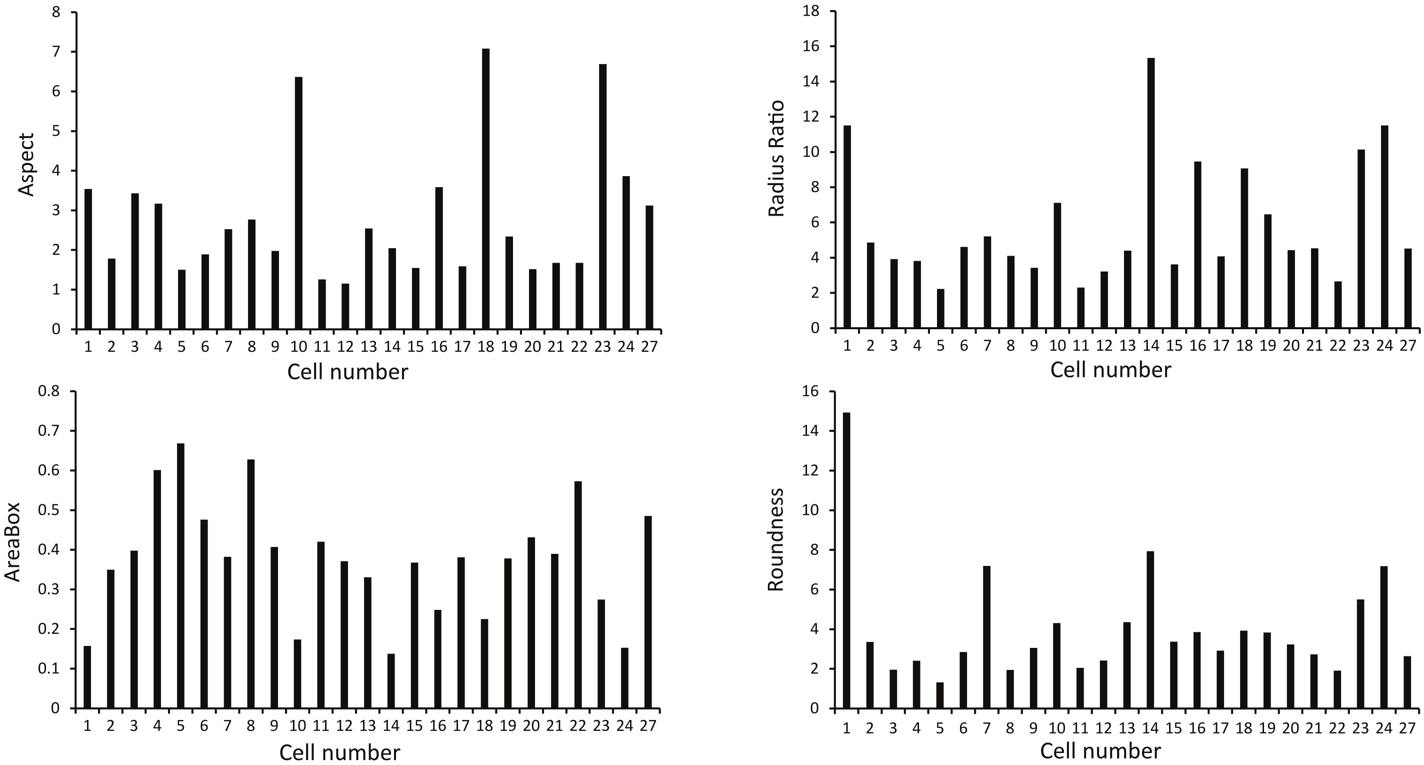

F

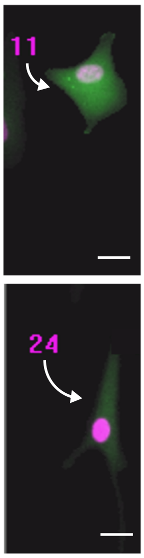

	Cell #11	Cell #24
**Area**	4252	4223
**Aspect**	1.25	3.85
**Area/Box**	0.42	0.15
**RadiusRatio**	2.31	11.5
**Roundness**	2.05	7.18
**CII**	5.19	22.38

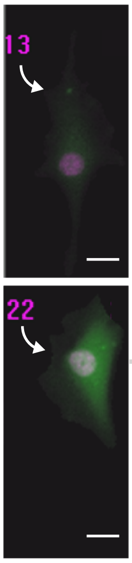

	Cell #13	Cell #22
**Area**	9182	8700
**Aspect**	2.54	1.67
**Area/Box**	0.33	0.57
**RadiusRatio**	4.40	2.65
**Roundness**	4.36	1.90
**CII**	10.97	5.65

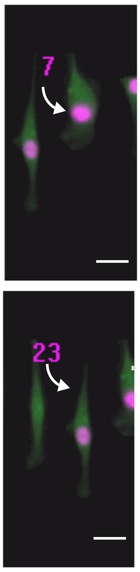

	Cell #7	Cell #23
**Area**	2045	2108
**Aspect**	2.53	6.69
**Area/Box**	0.38	0.27
**RadiusRatio**	5.22	10.13
**Roundness**	7.20	5.50
**CII**	14.57	22.05

Supp. Figure 2 – Representative examples of CellMorph variables in individual cells in control condition. (A) Cellmorph image analysis parameters used into CII equation explained. (B) Correlation matrix for shape variables. (C) Representative examples of cells from the control condition (U87 GFP-LC3, mAppletrunc-53BP1); scale bar: 20µm. Green fluorescence: GFP-LC3; red fluorescence: mAppletrunc-53BP1. (C) Area of cells shown in B. (D) Individual shape measurements for the cells shown in B. (E) Comparison of the primary Cell Morph variables between pairs of cells shown in B with similar cellular areas, to allow comparative observation of measurements in cells with different phenotypes; scale bar: 10µm.

A

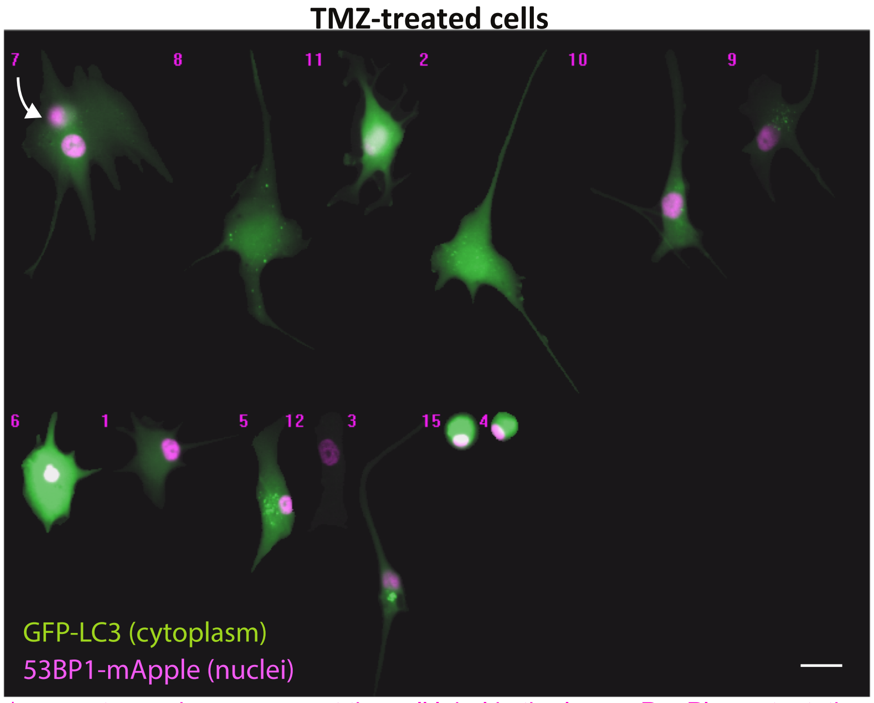

* magenta numbers represent the cell label in the Image Pro Plus output; the cell's label corresponds to the number to its left, as exemplified in the first cell.

B

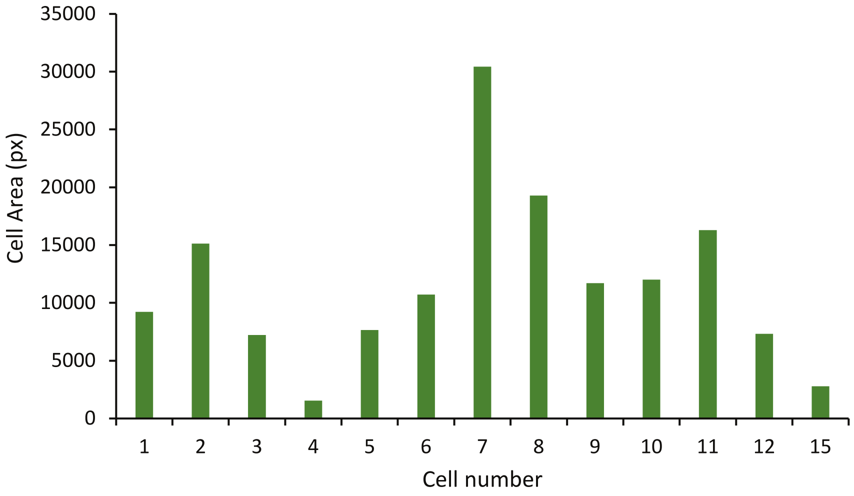

C

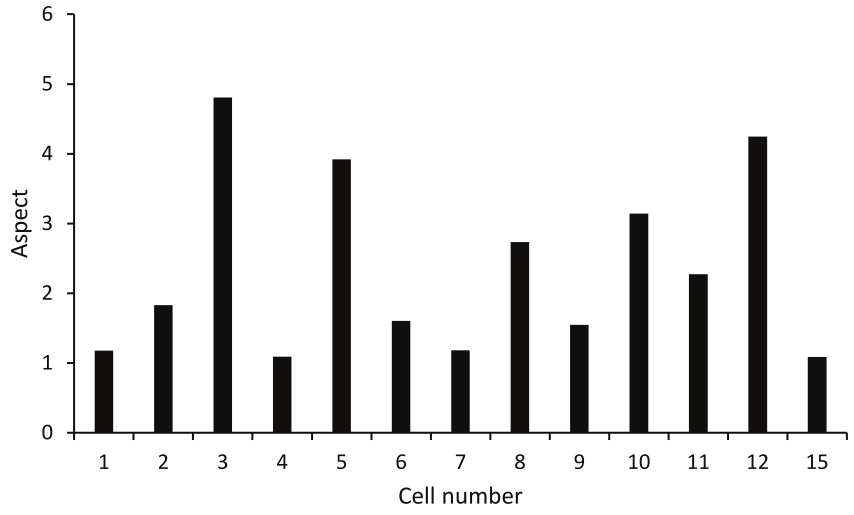

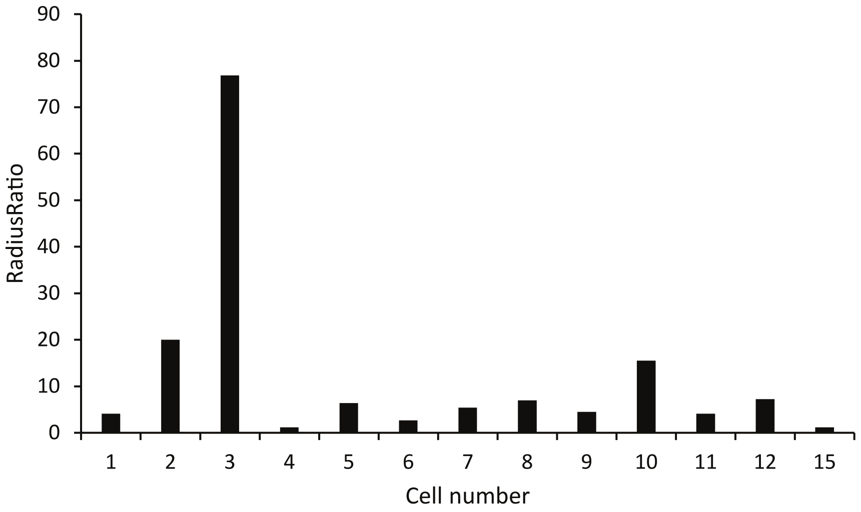

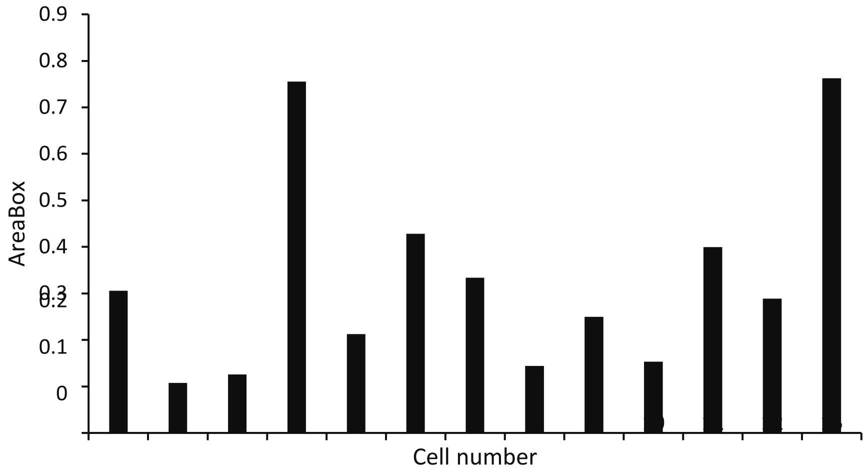

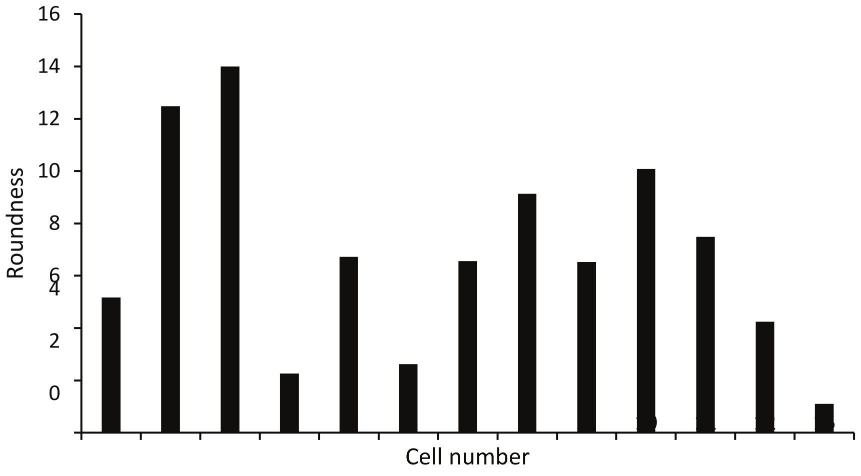

D

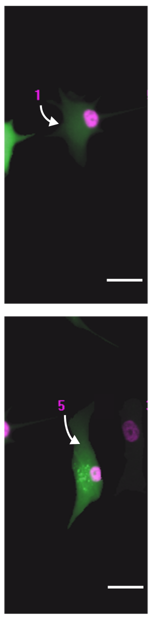

	Cell	
	#1	#5
Area	9225	7640
Aspect	1.18	3.92
Area/Box	0.31	0.21
RadiusRatio	4.14	6.42
Roundness	5.17	6.73
CII	10.18	16.86

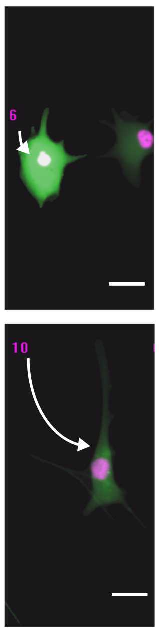

	Cell	
	#6	#10
Area	10710	12021
Aspect	1.60	3.14
Area/Box	0.43	0.15
RadiusRatio	2.66	15.52
Roundness	2.62	10.08
CII	6.45	28.59

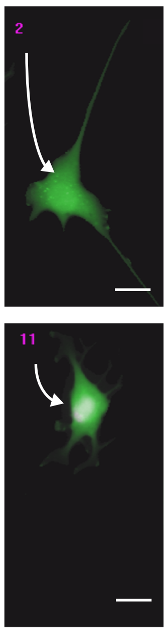

	Cell	
	#2	#11
Area	15123	16285
Aspect	1.83	2.27
Area/Box	0.11	0.40
RadiusRatio	20.03	4.09
Roundness	12.48	7.49
CII	34.23	13.45

Supp. Figure 3 - Representative examples of CellMorph variables in individual cells after TMZ treatment. (A) Representative examples of cells from the TMZ-treated condition (U87 GFP-LC3, mApple-53BP1); scale bar: 20µm. Green fluorescence: GFP-LC3; red fluorescence: mAppletrunc-53BP1. (B) Area of cells shown in A. (C) Individual shape measurements for the cells shown in A. (D) Comparison of the primary Cell Morph variables between pairs of cells shown in A with similar cellular areas, to allow comparative observation of measurements in cells with different phenotype; scale bar: 20µm.

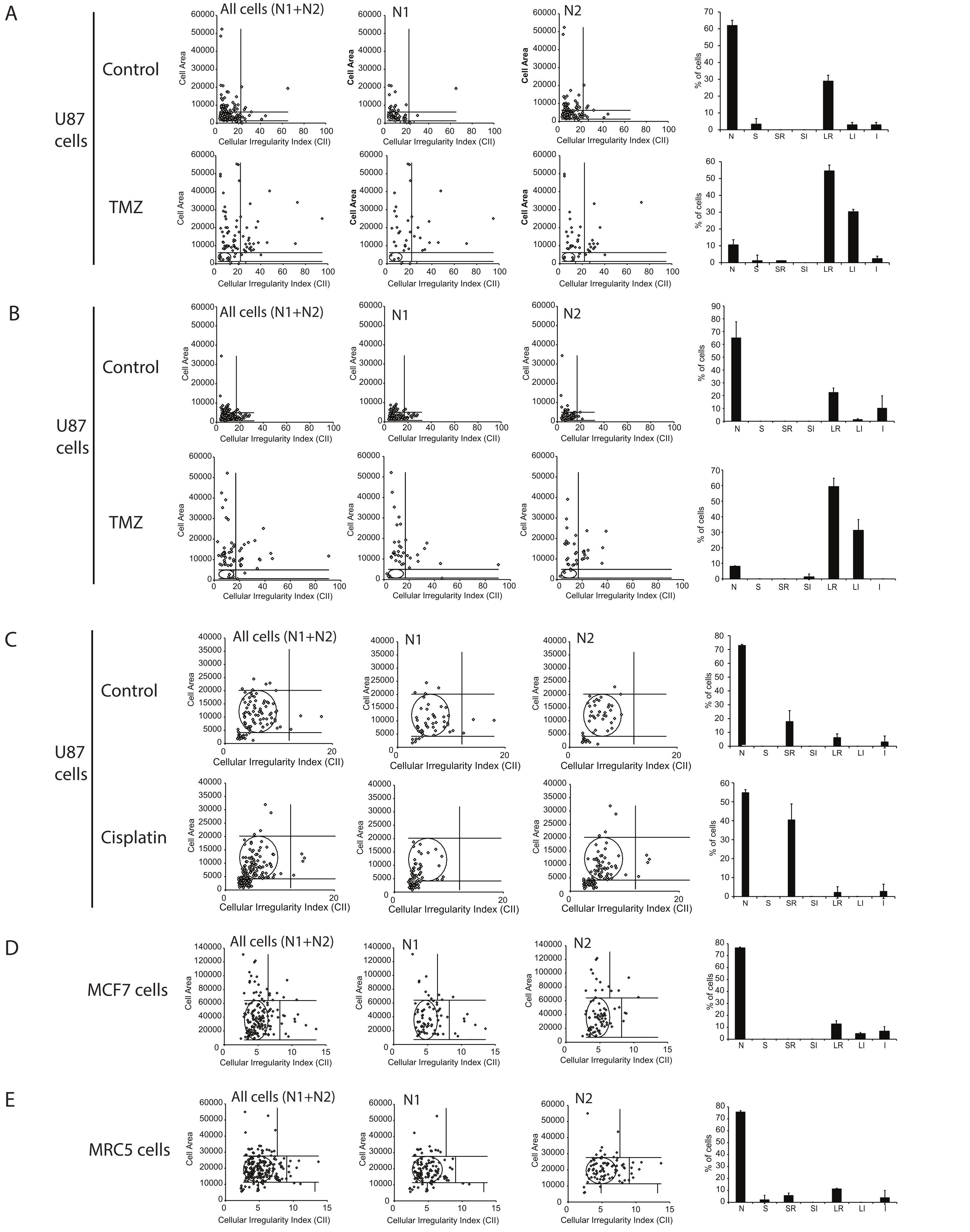

Supp. Figure 4 - Differential distribution of cells in the CellMorph plot for different experimental replicates. The first column shows the set of all cells, while the second and third columns indicate the distribution for each experimental replicate. The right column shows the distribution in the quadrants of the CellMorph plot. Five models used in the study are shown. (A) CellMorph (bright-field) for U87 cells (control and TMZ-treated). (B) CellMorph fluorescence for U87 cells (control or treated with TMZ 50uM for 5d). (C) CellMorph (bright-field) for U87 cells (control or treated with cisplatin 20uM for 24h). (D) MCF7 cells. (E) MRC5 cells.

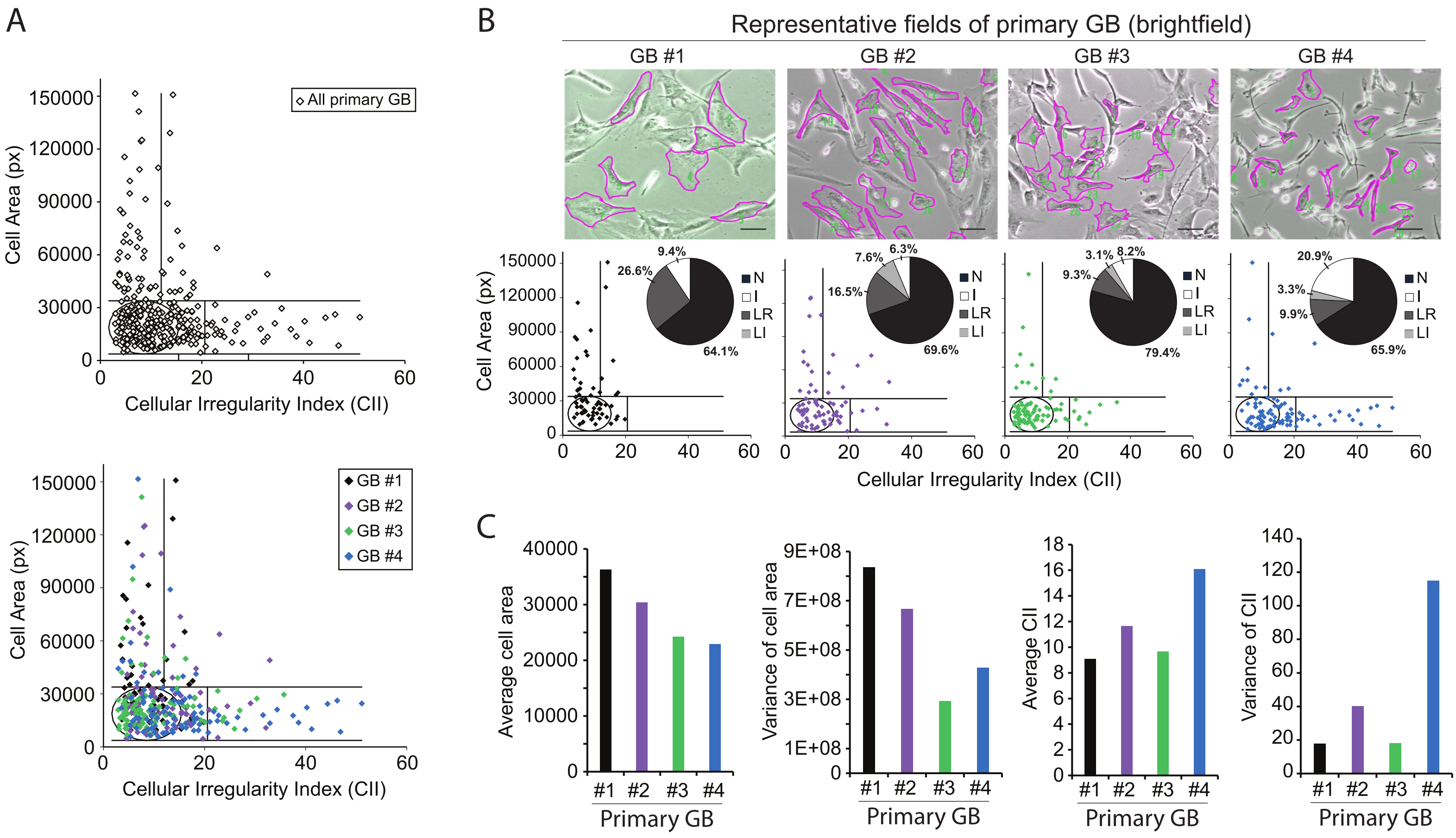

Supp. Figure 5 - CellMorph applied to primary culture. Four primary glioblastoma (GB) cells were isolated and imaged. (A) Top - CellMorph scatter plot. All primary cell cultures are shown together; bottom – individual primary cultures; patients are demonstrated with markers of different colors. GB#1, n=66 cells; GB#2, n=81 cells; GB#3, n=99 cells; GB#3, n=93 cells; (B) Representative images of each primary cell culture with their respective CellMorph scatterplots and distribution of cells in each quadrat (pie chart); scale bar: 10µm. C) Average and variance measurements of Cell Area and CII for each primary culture.

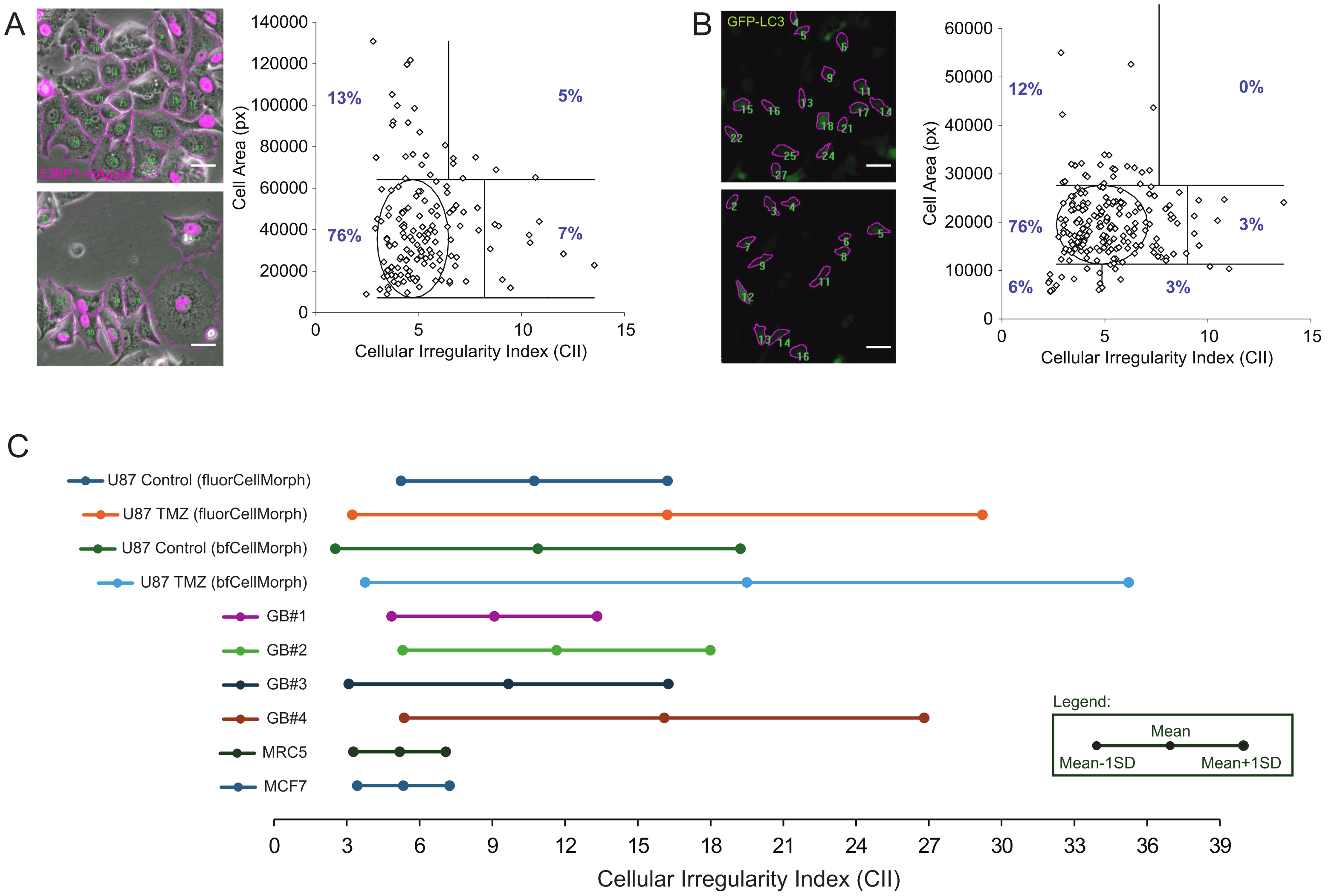

Supp. Figure 6 - CellMorph applied to MCF7 epithelial breast cancer cells and MRC5 fibroblasts. (A) Two representative images of cellular segmentation (left) and CellMorph scatterplot (right) for MCF7 breast cancer cells; n=150 cells; scale bar: 20µm. (B) Two representative images of cellular segmentation (left) and CellMorph scatterplot (right) for MRC5 GFP-LC3 human fibroblasts; n=209 cells; scale bar: 20µm; (C) CII range for the different models used in the standardization and validation of CellMorph.

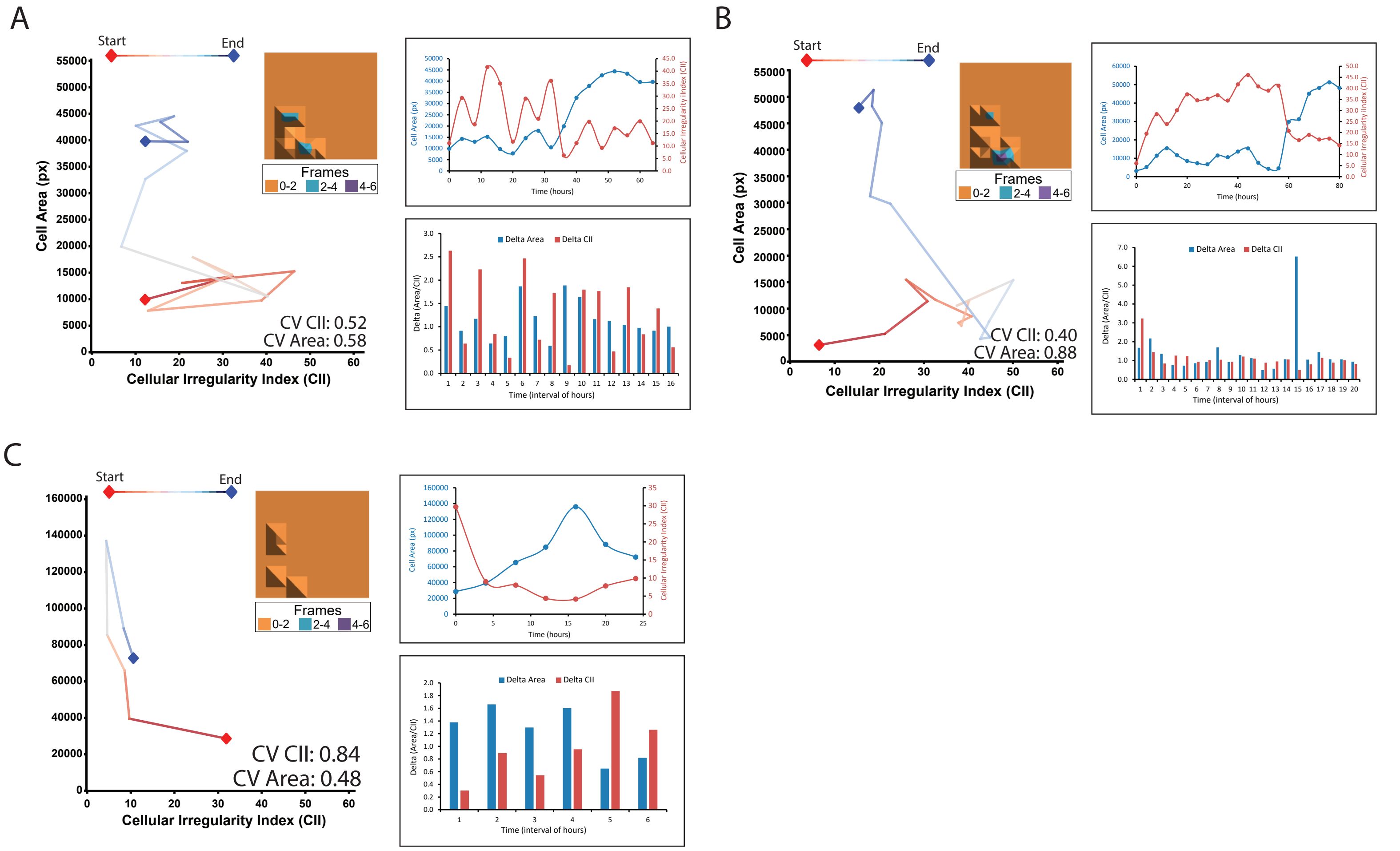

Supp. Figure 7 – Examples of trackingCellMorph. (A-C) Three additional examples of individual cells with different profiles of morphometric variability. To each cell, the trackingCellMorph scatterplot and density plots are shown. Red and blue markers indicate the phenotypic state at the start and end of tracking, respectively. We also added the coefficient of variance (CV) information for Cell Area and CII. On the right, line graphs of cell area and CII over time are also shown (top), as well as a bar graph with deltas of cell area and CII in each measurement interval (bottom).

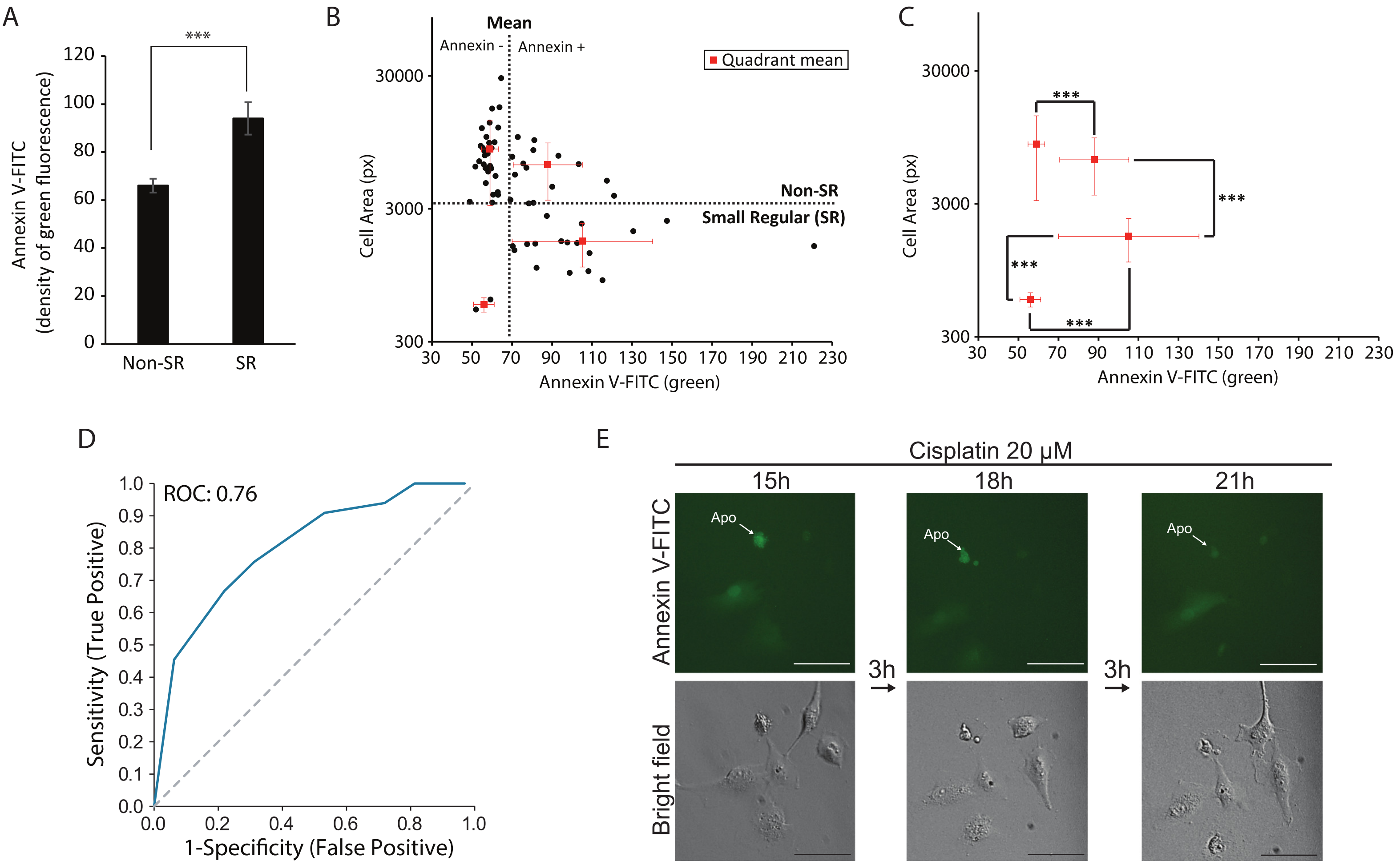

Supp. Figure 8 - Relationship between annexin staining and cell size for studying apoptosis. (A) Comparative green fluorescence density between SR and non-SR cells. (B) Cell Area and annexin V-FITC staining intensity. The horizontal dashed line shows the separation between small regular (SR) cells and non-SR cells. The vertical dashed line represents the separation between annexin V-FITC positive and negative cells based on the median. (C) Phenotypic progression of apoptosis based on the difference in mean cell area and annexin staining. (D) ROC curve for cell area and annexin staining. (E) Tracking individual cells with Annexin V-FITC labeling. U87 MG-wt cells were treated with Cisplatin 20 μM. From 15h of treatment onwards, images were taken every 3h. White arrows indicate a cell with apoptotic morphology and labeling with annexin V-FITC; scale bar: 20μm.

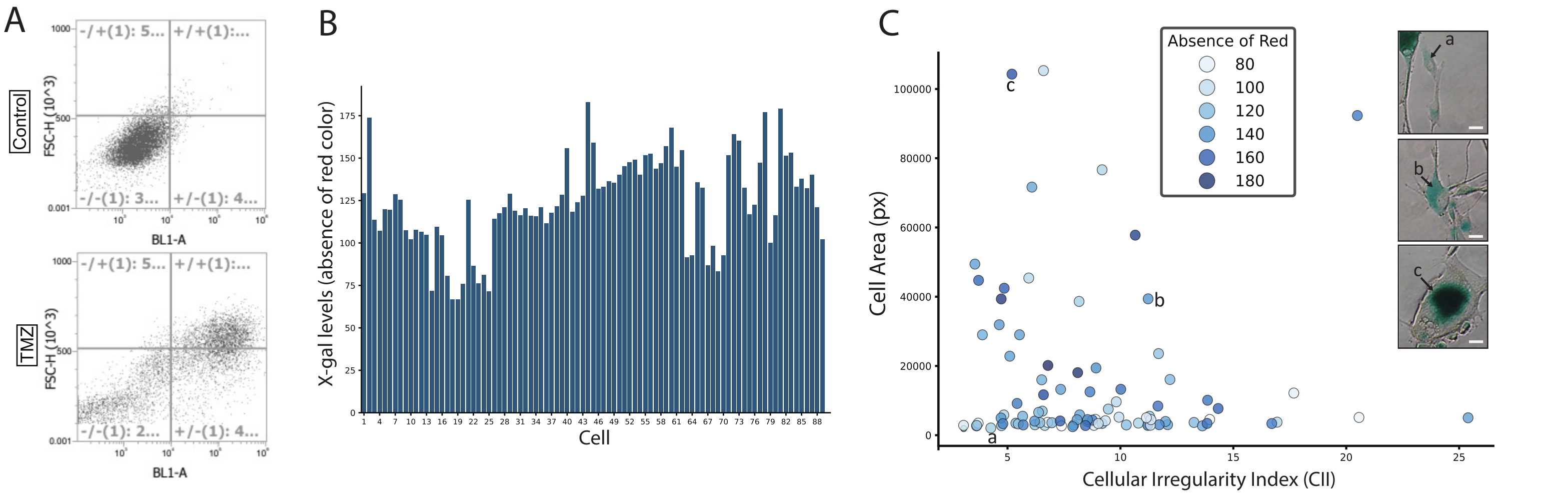

Supp. Figure 9 - CellMorph applied to study hypoxia-induced senescence. (A) Flow cytometry for FSC (cell size) versus C12-FDG (activity of SA-β-gal) in control and TMZ-treated conditions. (B and C) U87 MG-wt cells were maintained in hypoxia for 24h, followed by 7d in normoxia. (B) Levels of SA-β-gal activity, measured by Absence of Red color in representative x-gal stained cells. (C) CellMorph multidimensional scatterplot integrating data from Cell Area, CII and x-gal levels (Absence of Red) in individual cells. Insert – representative cells indicated in the scatterplot; scale bar: 10μm.

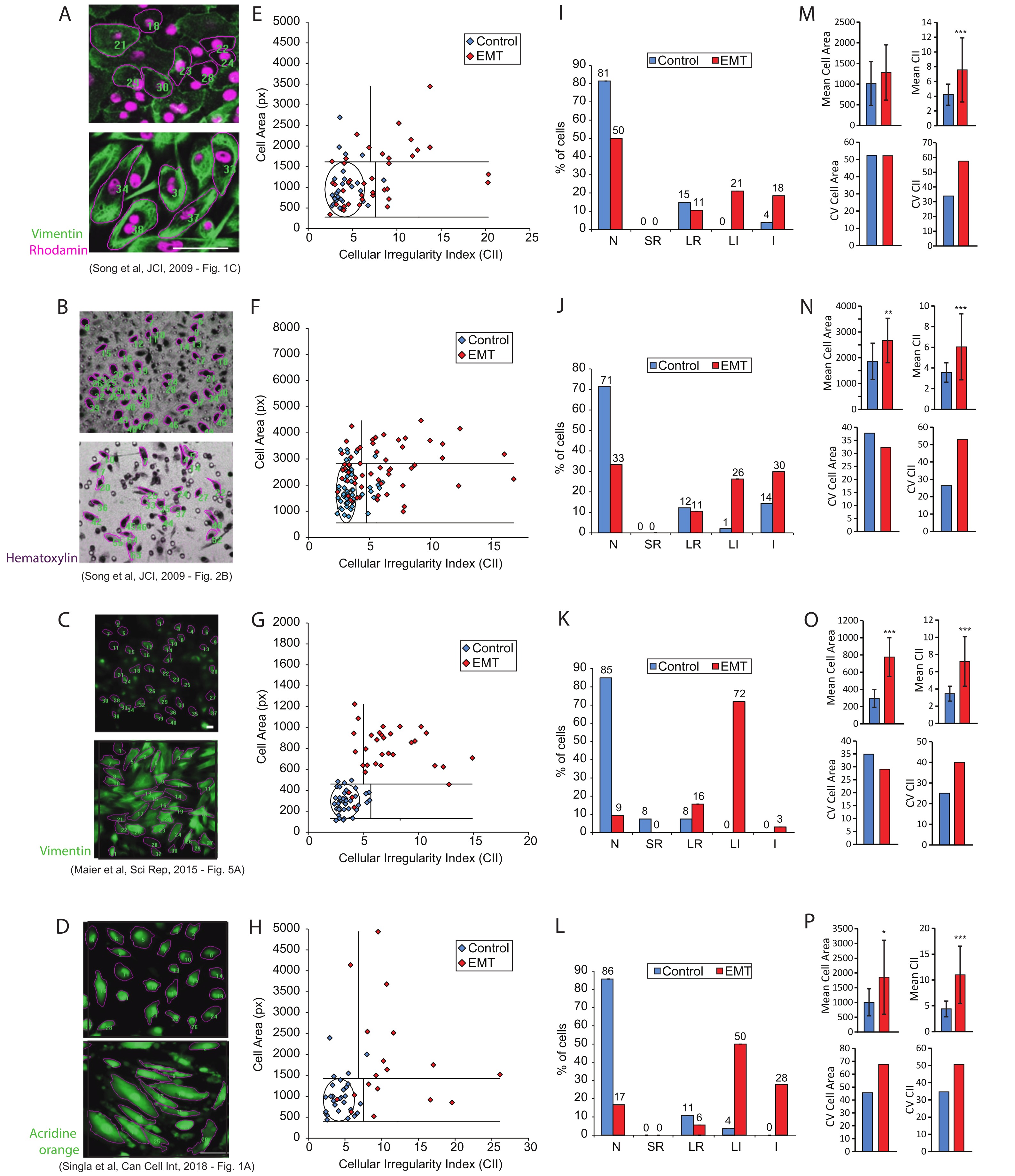

Supp Figure 10 – Validation of CellMorph to study epithelial-to-mesenchymal transition (EMT). (A-D) Figures obtained from the indicated references, showing the cell segmentation carried out to obtain measurements of cell area and shape. A, scale bar: 80µm; B, original magnification 400x; C, scale bar: 20µm; D, scale bar: 20µm. (E-H) Composed CellMorph scatterplots to each example, showing control (blue) and EMT (red) conditions. (I-L) Bar graphs showing the percentage of cells in each quadrant of CellMorph for control and EMT conditions. (M-P) Average and coefficient of variance (CV) measurements of Cell Area and CII to each example. *p<0.05, **p<0.01, ***p<0.001.

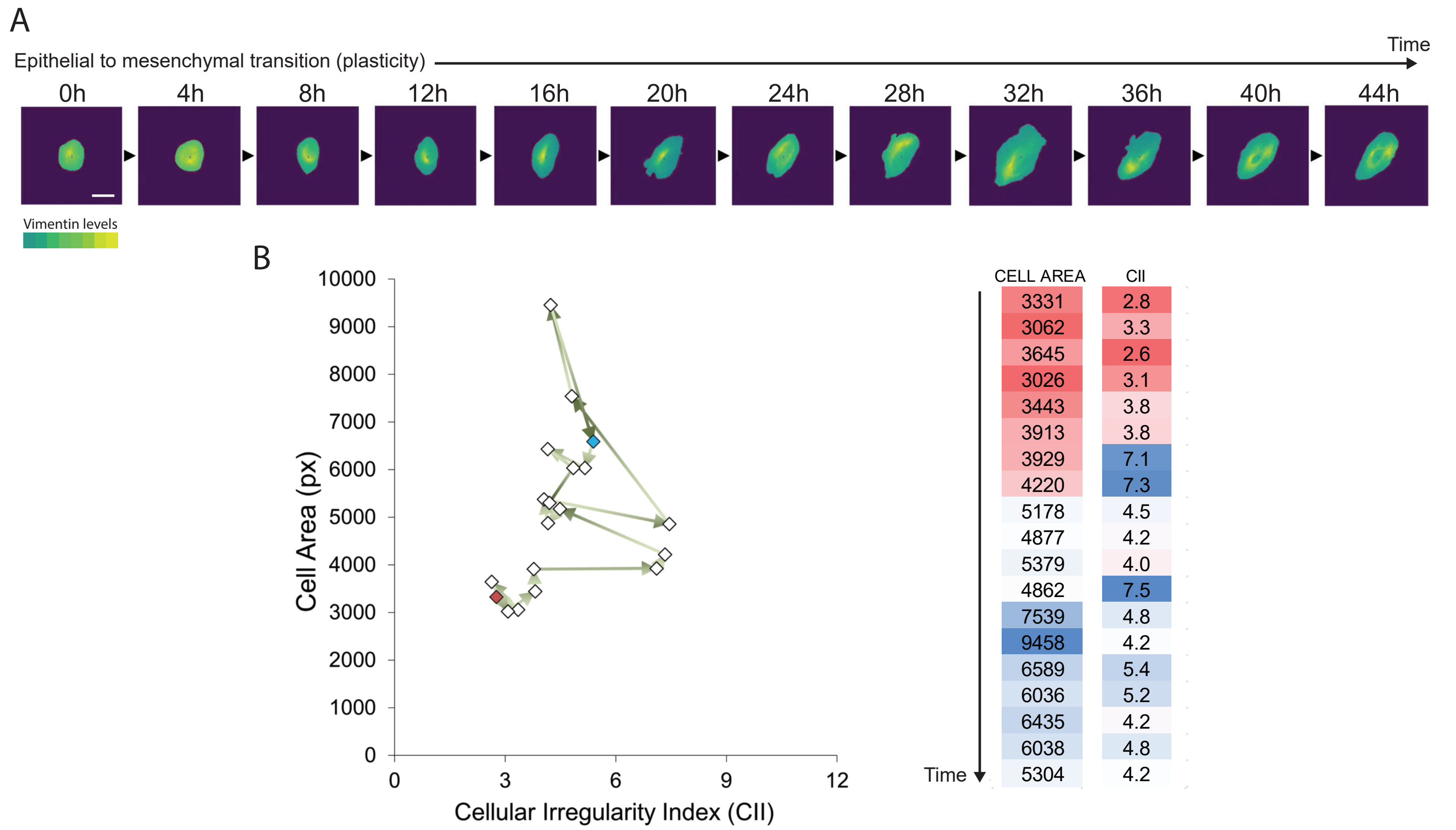

CELL AREA	CII
3331	2.8
3062	3.3
3645	2.6
3026	3.1
3443	3.8
3913	3.8
3929	7.1
4220	7.3
5178	4.5
4877	4.2
5379	4.0
4862	7.5
7539	4.8
9458	4.2
6589	5.4
6036	5.2
6435	4.2
6038	4.8
5304	4.2

Supp. Figure 11 - Validation of trackingCellMorph to study the cellular morphometric variation during epithelial-to-mesenchymal transition (EMT). The frames for segmentation were extracted from Video 1 ('Recorded live cell trajectory') of reference [42]; scale bar: 20µm. (A) Twelve frames of an individual cell that was segmented over time. (B) Left: TrackingCellMorph scatterplot for a cell during EMT. Red and blue markers indicate the phenotypic state at the start and the end of tracking. respectively. Right: Cell Area and CII values over time for the monitored cell. Color scale from red to green indicates the lower to higher values.

Supplementary table 1 – Shape measurements acquired in each cell segmentation software used.

**Image Pro Plus**	**Fiji**	**CellProfiler**	**Napari***
Aspect	Aspect Ratio (AR)	AreaShape_MajorAxisLength/AreaShape_MinorAxisLength	Aspect
Area / box	Area / (Width × Height)	AreaShape_Extend	Area / Box
Radius Ratio	Min Feret / Feret	AreaShape_MaxFeretDiameter/AreaShape_MinFeretDiameter	Radius Ratio
Roundness	1/ Circularity	1 / AreaShape_FormFactor	Roundness

*The parameters are determined and defined (including naming) by the user; we suggest the same names as described in Image Pro Plus for convenience.

Supplementary table 2 – significance of the difference between the shape variables and CII for the models used for the development and validation of CellMorph (p-values, Mann-Whitney).

	Aspect	AreaBox	RadiusRatio	Roundness	CII
**U87 cells C x TMZ bf**	8.82E-01	1.20E-07	1.09E-03	2.65E-08	6.83E-06
**U87 cells C x TMZ fluor**	3.78E-06	1.20E-07	1.09E-03	3.00E-08	5.87E-12
**Macrophages C x LPS**	1.24E-01	1.11E-06	4.37E-01	9.57E-01	1.00E-04
**C x EMT cells (Fig. S8A)**	1.05E-05	1.34E-03	1.68E-04	1.97E-04	1.91E-04
**C x EMT cells (Fig. S8B)**	3.03E-07	1.54E-07	2.18E-06	1.28E-06	4.65E-07
**C x EMT cells (Fig. S8C)**	2.31E-06	2.50E-04	1.62E-08	3.12E-09	2.57E-08
**C x EMT cells (Fig. S8D)**	2.82E-05	1.80E-03	1.35E-03	7.77E-04	4.40E-05
**Mean**	1.44E-01	4.85E-04	6.29E-02	1.37E-01	4.89E-05

Supplementary table 3 – Correlation of cell area and CII to all models used in CellMorph development and validation.

Model	Cell Area and CII correlation (Pearson factor)
U87 C (fluorescence)	-0.192
U87 TMZ (fluorescence)	-0.161
U87 C (brightfield)	-0.039
U87 TMZ (brightfield)	-0.091
MCF7	0.006
MRC5	0.083
Primary GB (GB1, GB2, GB3, GB4)	-0.066 / -0.082 / -0.030 / -0.206
EMT (Fig S8A - fluorescence) - C and EMT	0.088 / 0.401
EMT (Fig. S8B - brightfield) C and EMT	0.104 / 0.191
EMT (Fig. S9C) - C and EMT	0.167 / 0.0309
EMT (Fig. S8D) - C and EMT	0.128 / -0.130
Macrophages - C (PBS) and LPS	0.099 / 0.523
